# IFITM1 as a modulator of surfaceome dynamics and aggressive phenotype in cervical cancer cells

**DOI:** 10.3892/or.2025.8904

**Published:** 2025-04-29

**Authors:** Nela Friedlová, Lucie Bortlíková, Lenka Dosedělová, Lukáš Uhrík, Ted R. Hupp, Lenka Hernychová, Bořivoj Vojtěšek, Marta Nekulová

**Affiliations:** 1 Research Centre for Applied Molecular Oncology, Masaryk Memorial Cancer Institute, 656 53 Brno, Czech Republic; 2 Department of Experimental Biology, Faculty of Science, Masaryk University, 625 00 Brno, Czech Republic; 3 University of Edinburgh, Institute of Genetics and Molecular Medicine, EH4 2XU Edinburgh, United Kingdom

**Keywords:** interferon-induced transmembrane proteins, surfaceome, cervical cancer, mass spectrometry, migration, invasion, membrane protein, antigen presentation, immunosurveillance

## Abstract

Interferon-induced transmembrane proteins (IFITMs) are frequently overexpressed in cancer cells, including cervical carcinoma cells, and play a role in the progression of various cancer types. However, their mechanisms of action remain incompletely understood. In the present study, by employing a combination of surface membrane protein isolation and quantitative mass spectrometry, it was comprehensively described how the IFITM1 protein influences the composition of the cervical cancer cell surfaceome. Additionally, the effects of interferon-γ on protein expression and cell surface exposure were evaluated in the presence and absence of IFITM1. The IFITM1-regulated membrane and membrane-associated proteins identified are involved mainly in processes such as endocytosis and lysosomal transport, cell-cell and cell-extracellular matrix adhesion, antigen presentation and the immune response. To complement the proteomic data, gene expression was analyzed using reverse transcription-quantitative PCR to distinguish whether the observed changes in protein levels were attributable to transcriptional regulation or differential protein dynamics. Furthermore, the proteomic and gene expression data are supported by functional studies demonstrating the impact of the IFITM1 and IFITM3 proteins on the adhesive, migratory and invasive capabilities of cervical cancer cells, as well as their interactions with immune cells.

## Introduction

Cervical cancer remains the fourth leading cause of cancer-related incidence and mortality, with 660,000 new cases and 350,000 deaths among women worldwide in 2022 (1). Despite ongoing advancements in disease management, patient prognosis is influenced by multiple individual factors, highlighting the urgent need for personalized therapeutic strategies guided by reliable prognostic and predictive biomarkers (2–4).

In this context, the cancer cell surface proteome (surfaceome) has emerged as a critical area of study due to its role in tumor progression and treatment response. Defined as the complete set of plasma membrane proteins with at least one amino acid residue exposed to the extracellular space (5), the surfaceome facilitates the selective movement of substances and serves as an anchoring base for the cytoskeleton, and its structure influences crucial signaling processes triggered by interactions between cells and the extracellular matrix. The surfaceome includes receptors, transporters, channels, cell-adhesion proteins and enzymes, making it a rich source of potential diagnostic biomarkers and important targets for therapeutic interventions.

Interferon-induced transmembrane proteins (IFITMs) are small Dispanin proteins containing two (trans) membrane helices that localize in the plasma membrane and endosomal/lysosomal membranes (6). Immunity-related IFITMs (IFITM1, IFITM2 and IFITM3) are highly homologous, but they differ in their subcellular localization and function, as demonstrated in recent years (7,8). IFITM1 differs from IFITM2 and IFITM3 by a 21-amino acid truncation at the N-terminus and a 13-amino acid extension at the C-terminus. Unlike IFITM2 and IFITM3, which are predominantly found in endosomal membranes, IFITM1 is localized primarily in the plasma membrane on the cell surface.

Immunity-related IFITMs are strongly induced by type I and type II interferons (IFNs) and function mainly as restriction factors that block viral infections. They also play an important role in cancer progression (6,9–11). Although the exact mechanisms of their function remain unknown, IFITMs influence membrane structure and function (12–14), the intracellular trafficking of endocytosed cargo (15), and pH regulation within the vesicular environment (16,17). These processes are crucial for regulating surface protein expression, trafficking and recycling, leading us to hypothesize that IFITM1 could modulate surfaceome composition.

The aim of the present study was to elucidate the mechanisms by which IFITMs contribute to tumor progression. Initial investigations revealed that the suppression of IFITM1 in cervical cancer cells affects the levels of members of the IFN-related DNA damage resistance signature (IRDS) (18), including major histocompatibility complex class I (MHC-I), suggesting a possible explanation for the inverse correlation between metastasis formation and IFITM1/3 expression in cervical cancer tissues. Building on these findings, the authors focused on the cell surface proteome and, for the first time to the best of our knowledge, it was demonstrated how IFITM1 levels impact the cervical cancer cell surfaceome and associated cell phenotype.

## Materials and methods

### Cell culture

SiHa cell line [cat. no. HTB-35; American Type Culture Collection (ATCC)] was cultured in RPMI-1640 medium (cat. no. R4130; MilliporeSigma) supplemented with 10% fetal bovine serum (cat. no. S-FBS-SA-015; Serana Europe GmbH) and penicillin-streptomycin (cat. no. XC-A4122; Biosera) at 37°C with 5% CO_2_. The cells were passaged every two days using Accutase (cat. no. A6964; MilliporeSigma). Cell line authentication (STR profiling) and mycoplasma testing were performed regularly.

The lentiviral CRISPR/Cas9 system was used to generate single- and double-IFITM1- and IFITM3-knockout (KO) cells. The generation and characterization of IFITM1- and IFITM3-KO SiHa clones were described previously (18). SiHa wild-type (wt) and SiHa scrambled cells served as controls. SiHa scrambled control was generated using the CRISPR/Cas method with sgRNA that lacks a complementary sequence in the genome. For all methods used to evaluate the effect of IFNγ, the cells were treated with human IFNγ recombinant protein (cat. no. PHC4031; Gibco™; Thermo Fisher Scientific, Inc.) at a concentration of 100 ng/ml for 24 h. SiHa cell line exhibits baseline IFN pathway activity, and the IFNγ concentration used ensures effective and measurable exogenous pathway stimulation, as confirmed by the detection of IFN-stimulated protein levels following treatment with increasing IFNγ concentrations.

### Pulse stable isotope labeling with amino acids in cell culture (SILAC), biotinylation and isolation of cell surface proteins

For pulse SILAC, SiHa wt and IFITM1 KO cells (clones no. I, II and IV) were cultured in biological triplicates for 24 h on 10-cm plates in R6K4 RPMI SILAC medium (cat. no. SM022; Gemini Biosciences) containing 13C-labeled arginine and 2D labeled lysine supplemented with dialyzed FBS (cat. no. A3382001; Gibco; Thermo Fisher Scientific, Inc.) and penicillin-streptomycin. Three independently created clones (no. I, II and IV) were used as biological replicates of IFITM1 KO cells to minimize the effect of clonal selection. The cells from 3×10 cm-well plates were harvested with Accutase, rinsed in ice-cold PBS and incubated with 0.8 mM cell impermeable EZ-Link Sulfo-NHS-SS-Biotin (cat. no. 21331; Thermo Fisher Scientific, Inc.) in PBS for 10 min. After centrifugation at 200 × g/4°C/5 min, 50 mM Tris (pH 7.5) was added to quench the excess Sulfo-NHS-SS Biotin. After washing in PBS, the cell pellets were suspended in lysis buffer (2% NP-40, 0.2% SDS, 10 mM EDTA, 108 mM oxidized glutathione, 1X protease inhibitor mix) and incubated on ice for 30 min. Biotinylated proteins were incubated with Pierce high-capacity streptavidin agarose (cat. no. 20359; Thermo Fisher Scientific, Inc.) for 1 h; washed 3 times in Buffer A (1% NP-40, 0.1% SDS, 5 mM oxidized glutathione), 2 times in Buffer B (10% SDS, 5 mM oxidized glutathione), 2 times in Buffer C (2 M NaCl, 0.1% SDS, 5 mM oxidized glutathione, heated to 40°C) and 4 times in Buffer D (50 mM Tris (pH 8), 5 mM oxidized glutathione); and then eluted with 200 mM DTT reducing agent in 125 mM Tris (pH 6.8) for 5 min at 85°C.

### MS analysis

#### Peptide generation using FASP

Proteins were digested using a filter-aided sample preparation protocol (FASP) (19). Briefly, whole material obtained from enriched cell surface proteins was loaded on Microcon Ultracel-30 spin filter columns with a 30-kDa cutoff (MilliporeSigma). Urea buffer (8 M urea in 0.1 M Tris, pH 8.5) was added to the filter column, which was subsequently centrifuged at 14,000 × g/20°C/15 min. Protein reduction was performed via the addition of 100 mM Tris (2-carboxyethyl) phosphine hydrochloride (MilliporeSigma) in urea buffer at 37°C/30 min/600 rpm in a thermomixer. The column was centrifuged at 14,000 × g/20°C/15 min, and free sulfhydryl groups were alkylated using 300 mM iodoacetamide in urea buffer (MilliporeSigma). Protein alkylation was performed in the dark for 20 min, followed by another centrifugation at 14,000 × g/15 min. Ammonium bicarbonate was added to the column (final concentration of 10 µM), followed by centrifugation at 14,000 × g/20 min. Trypsin (Promega Corporation) was added at a 1:100 ratio, and the proteins were digested overnight at 37°C. The columns were centrifuged at 14,000 × g/15 min, and the peptides were desalted on C18 Micro spin columns (Harvard Apparatus).

### LC-MS/MS analyses

The samples were separated using an UltiMate 3000 rapid separation liquid chromatography (RSLC) nano chromatograph (Thermo Fisher Scientific, Inc.). Peptides were loaded on a precolumn (µ-precolumn, 30 m i.d., 5 mm length, C18 PepMap 100, 5 µm particle size, 100 Å pore size) and further separated on an Acclaim PepMap RSLC column (75 µm i.d., length 250 mm, C18, particle size 2 µm, pore size 100 Å) with a flow rate of 300 nl/min using a linear gradient of B (80% acetonitrile in 0.08% aq. formic acid) in A (0.1% aq. formic acid): 2% B for 4 min, 2–40% B for 64 min, 40–98% B for 2 min, with A (0.1% aq. formic acid) and B (80% acetonitrile in 0.08% aq. formic acid). Peptides eluted from the column were introduced into an Orbitrap Elite mass spectrometer (Thermo Fisher Scientific, Inc.) operating in the Top20 data-dependent acquisition mode. A survey scan of 400–2,000 m/z was performed at 120,000 resolution with an AGC target of 1×10^6^ and a 200 ms injection time, followed by 20 data-dependent MS2 scans performed in the LTQ linear ion trap with 1 microscan, a 10 ms injection time and 10,000 AGC.

### Database searching and data evaluation

The mass spectrometry data were processed using Proteome Discoverer (Thermo Fisher Scientific, Inc.), version 2.2, for SILAC-labeled samples. For the raw files of each triplicate sample set, a consensus workflow was applied, consisting of the following sequential nodes: the Minora feature detector, the Precursor Ion Quantifier, and the Feature Mapper. Data processing was performed using the Sequest HT engine with the following search settings: database Swiss-Prot TaxID=9606, 2017-10-25, sequences 42252, taxonomy: Homo sapiens (updated September 2019) and database with contaminants: PD_Contaminants_2015_5fasta; enzyme trypsin; 2 missed cleavage sites; precursor mass tolerance 10 ppm; fragment mass tolerance 0.6 Da; static modification carbamidomethyl [+57.02 Da, (C)], label 13C(6) [+6.020 Da (R)] and label 2H(4) [+4.025 Da (K)]; dynamic modifications oxidation [+15.995 Da (M)], Met-loss + Acetyl [protein terminus, −89.030 Da (M)]. The results were further validated using the Percolator algorithm (version integrated in Proteome Discoverer 2.2), which applies semi-supervised machine learning for peptide-spectrum match validation and false discovery rate (FDR) estimation, with a FDR threshold of 1%. For the protein quantification and statistical assessment of the biological triplicates, the Precursor Ions Quantifier was applied with the following parameters: using only unique peptides and razor peptides, normalization on total peptide amount, pairwise ratio calculation and ANOVA (background-based) hypothesis test. The relative quantification value is represented as the relative peak area of the peptides with heavy isotope labels in SiHa wt cells compared with IFITM1 KO cells or cells treated with IFNγ compared with control SiHa cells without any treatment (Appendix S1). The normalized peptide abundances were used to calculate the statistical significance of differences between groups (wt vs. IFITM1 KO; untreated vs. IFNγ-treated samples) using an unpaired two-tailed t-test.

Gene Ontology (GO) analysis was performed using DAVID Bioinformatics Resources 6.8 (20), and protein-protein interactions were mapped using STRING database (21). The mass spectrometry proteomics data have been deposited in the ProteomeXchange Consortium via the PRIDE repository (22) with the dataset identifier PXD053951 and the following access login credentials: Username: reviewer_pxd053951@ebi.ac.uk; Password: ouMaYQI4Qg6T.

### Flow cytometry

The cells were harvested using Accutase, washed in 1% BSA (cat. no. A3059; MilliporeSigma) in PBS, and incubated with PE-conjugated anti-CD166 monoclonal antibody (cat. no. 12-1668-42) or FITC-conjugated anti-CD40 monoclonal antibody (cat. no. 11-0409-42; both from Invitrogen; Thermo Fisher Scientific, Inc.) for 30 min on ice in the dark. After a second wash, the cells were resuspended in 1% BSA in PBS and measured with a FACSVerse instrument (BD Biosciences). The results were analyzed using FACSSuite (BD Biosciences). Statistical analysis was performed using an unpaired t-test with data from three biological replicates.

### Protein extraction, PAGE and western blotting

The cells were scraped into 1X LDS lysis buffer on ice: 105 mM Tris hydrochloride, 140 mM Tris base, 75 mM LDS, 0.5 mM EDTA, 10% glycerol, 1X protease inhibitor cocktail (cat. no. 78429; Thermo Fisher Scientific, Inc.), and 0.1 mM PMSF (Thermo Fisher Scientific, Inc.). The samples were frozen after lysis. The cell lysates were sonicated and cleared by centrifugation (20,000 × g/30 min/4°C). The total protein concentration was measured using a DC protein assay (cat. no. 5000112; Bio-Rad Laboratories, Inc.). Appropriate amounts of cell lysates were mixed with DTT (MilliporeSigma) to a final concentration of 100 mM and 4X NuPAGE LDS Sample Buffer (cat. no. NP0008; Invitrogen; Thermo Fisher Scientific, Inc.) and then heated at 95°C for 10 min. Samples (20 µg/lane) were loaded into 10% polyacrylamide gels, separated by molecular weight, and transferred onto nitrocellulose membranes using the Mini-PROTEAN system (Bio-Rad Laboratories, Inc.). The membranes were blocked in 5% non-fat milk in phosphate-buffered saline with 0.1% Tween 20 (PBST) at room temperature for 1 h and incubated with primary antibodies diluted in milk buffer overnight at 4°C. After washing in PBST, the membranes were incubated at room temperature for 1 h with HRP-linked secondary antibodies. After washing in PBST and PBS, the chemiluminescent signal was visualized using enhanced chemiluminescence (ECL) reagents and a G:BOX Chemi XRQ image analysis system and software (Syngene). Fiji ImageJ software v. 2.14.0/1.54f (National Institutes of Health) (23) was used to quantify the signal intensity of individual proteins relative to the intensity of the loading control β-actin.

The primary antibodies and their dilutions used were as follows: anti-IFITM1 (1:1,000; 5B5E2; cat. no. 60074-1-Ig; Proteintech Group, Inc.), anti-IFITM2 (1:500; 3D5F7; cat. no. 66137-1-Ig; Proteintech Group, Inc.), anti-IFITM3 (1:1,000; D8E8G; cat. no. 59212; Cell Signaling Technology, Inc.), anti-CD166 (1:1,000; EPR2759(2); cat. no. ab109215; Abcam) and anti-β-actin (1:5,000; cat. no. MA1-140; Invitrogen; Thermo Fisher Scientific, Inc.).

The secondary antibodies used were as follows: anti-rabbit IgG (1:2,000; cat. no. 7074; Cell Signaling Technology, Inc.) and anti-mouse IgG (1:5,000; Jackson ImmunoResearch Laboratories, Inc.).

### Migration and invasion assays

For the wound healing assay, cells (7×10^5^) were seeded into 12-well plates and incubated in complete growth medium for 48 h. To inhibit cell proliferation and avoid serum-free starvation conditions, the cells were treated with mitomycin C (10 µg/ml; M5353; MilliporeSigma) and incubated for 2 h at 37°C (24). The cells were then scratched and washed twice with PBS. To observe cell migration, the cells were cultivated in complete growth medium, and images were captured at time 0 (t_0_) and after 24 h (t_24_) using an Eclipse Ti-E inverted microscope (Nikon Corporation) at ×100 magnification. Fiji ImageJ software (23) with the Wound healing size tool plugin (25) was used for scratch area quantification. The migration rate (%) was calculated as [(scratch area t_0_-scratch area t_24_)/scratch area t_0_)]*100. The assay was repeated in biological triplicate (each in technical duplicate), and statistical analysis was conducted using an unpaired t-test.

For the Transwell migration assay, a 1×10^5^ cell suspension in serum-free RPMI was placed in the top chamber of each insert (cat. no. 140620; Thermo Fisher Scientific, Inc.). Complete RPMI medium was added to the lower chambers. The cells were incubated at 37°C for 24 h. After incubation, the cells on the upper surface of the inserts were removed, and the membranes were fixed with 70% ethanol at room temperature for 15 min. The migratory cells were stained with 0.5% crystal violet for 15 min. Images of the stained cells were captured using an Eclipse Ti-S microscope (Nikon Corporation). The number of migratory cells was calculated as an average of 5 fields of view (100× magnification) in each of the independent triplicates, and statistical analysis was conducted using an unpaired t test.

To evaluate the invasion potential of the cells, a 3D Matrigel drop invasion assay was performed as established by Aslan *et al* (26). Images were captured 6 days after drop formation using an EVOS™ FL Digital Inverted Microscope (Invitrogen™; objective magnification, ×4), and the invasion area (mm^2^) per field of view was calculated using Fiji ImageJ software (23). Two independent fields of view were evaluated for each drop. The assay was repeated in independent biological duplicates, each in technical triplicates, and statistical analysis was conducted using an unpaired t-test.

### Cervicosphere formation assay

Cells were seeded in ultralow attachment plates (Corning, Inc.) at a density of 10,000 viable cells per well using a FACSAria III. The cells were cultured in K-SFM (Gibco; Thermo Fisher Scientific, Inc.) supplemented with B27 (Gibco; Thermo Fisher Scientific, Inc.), 10 ng/ml human epidermal growth factor (MilliporeSigma) and 10 ng/ml bFGF (Gibco; Thermo Fisher Scientific, Inc.). The cells were then cultivated for 10 days. Images of the formed spheres were captured using an EVOS™ FL Digital Inverted Microscope (Invitrogen™; Thermo Fisher Scientific, Inc.; objective magnification, ×4), and the total number of spheres per well was determined. The sphere size was evaluated using Fiji ImageJ software (23) with a lower threshold of 6,000 µm^2^. The assay was repeated in independent biological triplicates, and statistical analysis was conducted using an unpaired t test.

### Reverse transcription-quantitative PCR (RT-qPCR)

Cells (1×10^6^) were seeded into 60 mm cell culture dishes and incubated overnight in complete RPMI. The cells were then treated with IFNγ (100 ng/ml; cat. no. PHC4031; Gibco; Thermo Fisher Scientific, Inc.) and further incubated for 24 h. RNA was isolated using the RNeasy Mini Kit (cat. no. 74104; Qiagen) and transcribed into cDNA using the High-Capacity cDNA Reverse Transcription Kit (cat. no. 4368814, Applied Biosystems™; Thermo Fisher Scientific, Inc.) following the manufacturer's protocol. Luna® Universal qPCR Master Mix (NEB) was used with 20 ng of cDNA per 10 µl reaction in a 384-well plate, along with specific primers (sequences below). Primers specific to *ACTB* were used as an endogenous control. The thermocycling conditions recommended by the master mix manufacturer were applied. qPCR was performed, and the results were analyzed using the QuantStudio 5 system (Applied Biosystems™; Thermo Fisher Scientific, Inc.). Relative gene expression was quantified using the ΔΔCq method (2^−ΔΔCq^) (27) from biological triplicates, each with technical triplicates. The SiHa wt control was chosen as the reference sample. Statistical analysis was conducted using the unpaired t-test to compare IFITM1 KO samples vs. scrambled ctrl (significance levels indicated by asterisks) and untreated (CTRL) vs. IFNγ-treated (IFNγ) samples (indicated by hashtags) separately. Primer sequences were as follows: IFITM1 forward, 5′-AGCACCATCCTTCCAAGGTCC-3′ and reverse, 5′-TAACAGGATGAATCCAATGGTC-3′; HLA-A forward, 5′-AAGTGGGAGGCGGCCCATGA-3′ and reverse, 5′-ATGTGTCTTGGGGGGGTCCGT-3′; HLA-B forward, 5′-ACTGAGCTTGTGGAGACCAGA-3′ and reverse, 5′-GCAGCCCCTCATGCTGT-3′; HLA-C forward, 5′-ATCGTTGCTGGCCTGGCTGTCCT-3′ and reverse, 5′-TCATCAGAGCCCTGGGCACTGTT-3′; IGF2R forward, 5′-TTGAGTGGCGAACGCAGTATGC-3′ and reverse, 5′-CAGTGATGGCTTCCCAGTTGTC-3′; M6PR forward, 5′-CTCCAGTCCCTCATCTCACC-3′ and reverse, 5′-AGCCTGCTACACCATTTTGC-3′; sortilin 1 (SORT1) forward, 5′-AGTTTCAGTGACCCACGTCAG and reverse, 5′-AGTAGGTCAGGTAACAAAGTCCAGT-3′; CD166/ALCAM forward, 5′-GAACACGATGAGGCAGACGA-3′ and reverse, 5′-TTCCATATTACCGAGGTCCTTGT-3′; CD40 forward, 5′-CTGGTCTCACCTCGCTATGG-3′ and reverse, 5′-GCAGTGGGTGGTTCTGGAT-3′; CD276 forward, 5′-GGAGAATGCAGGAGCTGAGG-3′ and reverse, 5′-GCCAGAGGGTAGGAGCTGTA-3′; and ACTB forward, 5′-GGAACGGTGAAGGTGACAGC-3′ and reverse, 5′-ACCTCCCCTGTGTGGACTTG-3′.

### Natural killer (NK) cytotoxicity assay

The NK-92 cell line purchased from ATCC (cat. no. CRL-2407) was cultured in Alpha Minimum Essential Medium without ribonucleosides and deoxyribonucleosides (cat. no. 22561021; Gibco; Thermo Fisher Scientific, Inc.) supplemented with 0.2 mM inositol (cat. no. I7508; MilliporeSigma), 0.1 mM 2-mercaptoethanol (cat. no. 21985023; Gibco; Thermo Fisher Scientific, Inc.), 0.02 mM folic acid (cat. no. F8758; MilliporeSigma), 200 U/ml recombinant IL-2 (cat. no. PHC0026; Gibco; Thermo Fisher Scientific, Inc.), 25% FBS and penicillin-streptomycin at 37°C with 5% CO_2_. The cells were passaged regularly every 2 days at a concentration of 2–3×10^5^ viable cells/ml. SiHa cells intended for cocultivation were washed and resuspended in PBS to a concentration of 1×10^6^ cells/ml. The cells were stained with CFSE solution (cat. no. 565082; BD Biosciences) at a final concentration of 2 µM in a 37°C water bath for 10 min. The cells were then washed thoroughly in PBS and complete RPMI. A total of 6×10^4^ cells per well were seeded in 24-well plates and allowed to grow overnight. The following day, complete RPMI was added to an NK-92 cell culture at a 1:1 ratio, and NK-92 cells were added at the indicated ratios to the CFSE-stained SiHa cells. The cell lines alone without coculture were used as controls. After 4 h of co-cultivation at 37°C with 5% CO_2_, SiHa cells were harvested with Accutase and mixed with collected medium containing NK-92 and dead SiHa cells. Dead cells stained with 7-AAD (cat. no. 559925; BD Biosciences) for 10 min were analyzed by FACSVerse (BD Biosciences). The dead SiHa cells were gated as the CFSE^+^ 7-AAD^+^ population (Figs. S1 and S3). Statistical analysis was performed using the unpaired t-test with data from three biological replicates.

### Statistical analysis

Statistical differences between two groups were analyzed using an unpaired two-tailed t-test. For proteomic data analysis, one-way ANOVA with background-based hypothesis testing (as implemented in Proteome Discoverer 2.2) was applied, followed by unpaired two-tailed t-tests for specific pairwise comparisons. Data is presented as the mean ± standard error of the mean (SEM). Statistical analysis and data visualization were performed using GraphPad Prism version 8.0.1 (GraphPad Software; Dotmatics). P<0.05 was considered to indicate a statistically significant difference.

## Results

### IFITM1 modulates the cervical cancer cell surfaceome in both IFNγ-dependent and IFNγ-independent manners

To investigate the IFITM1-regulated cancer cell surfaceome, we used the SiHa cervical cancer cell line, which expresses relatively high levels of IFITM1 protein even without IFN stimulation. In a previous study, it was demonstrated by the authors that IFITM1 regulates the surface expression of human leukocyte antigen B (HLA-B) in this cell line (18). IFITM1 KO SiHa cells were generated using CRISPR/Cas9 gene editing, and their preparation and characterization were performed as described previously (18).

**Figure 1.**
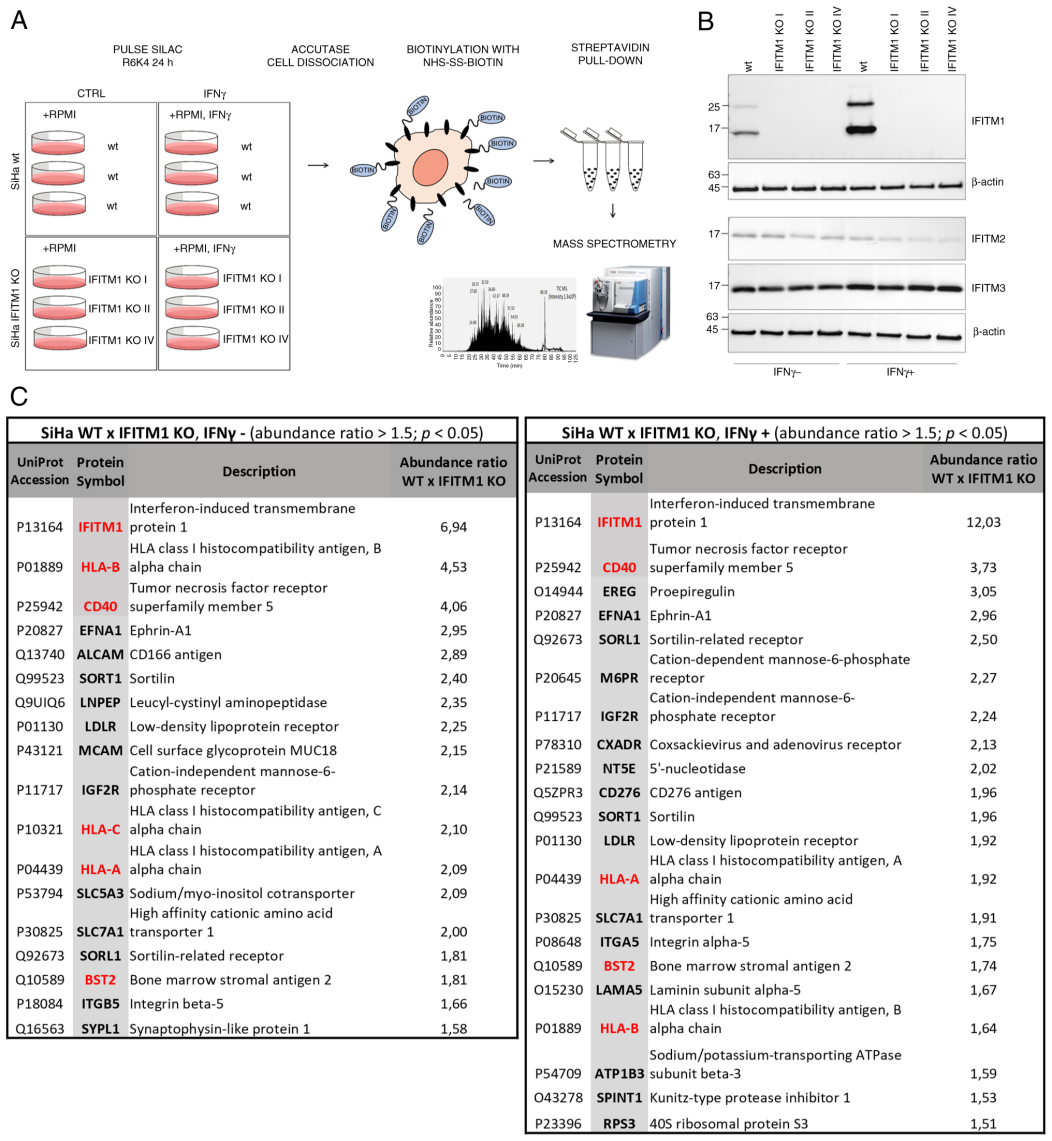
Surfaceome analysis of the IFITM1-deficient SiHa cervical cancer cell line. (A) A scheme of surface protein isolation, identification and quantification. (B) Western blot analysis of IFITM1, IFITM2 and IFITM3 proteins in SiHa wt and IFITM1 KO cells with or without IFNγ treatment. β-actin was used as a loading control. The upper band of IFITM1 (~25 kDa) corresponds to a potential dimer or a post-translationally modified IFITM1. (C) Membrane proteins identified by MS with a fold change in the protein abundance ratio wt/IFITM1 KO ≥1.5 (P<0.05) among untreated (left table) and IFNγ-treated cells (right table). Proteins with a fold change in the ratio of protein abundance ≥1.5 (P<0.05) in IFNγ-treated/untreated cells are highlighted in red. IFITM, interferon-induced transmembrane protein; IFN, interferon; SILAC, stable isotope labeling with amino acids in cell culture; wt, wild-type; KO, knockout.

To identify and quantify cell surface proteins whose levels differ between SiHa wt and IFITM1 KO cells, a proteomic approach was employed, combining SILAC, membrane protein isolation, and MS analysis (Fig. 1A). To capture proteins regulated only after IFN induction of IFITM1, cells were treated with IFNγ alongside SILAC medium, allowing protein turnover analysis. To minimize clonal selection bias, three independently generated IFITM1 KO cell clones were used as biological replicates (Fig. 1B). Western blot analysis of IFITM1 levels revealed an additional higher-molecular-weight band (~25 kDa) alongside the expected 15-kDa band. This band likely represents an IFITM1 dimer or a post-translationally modified form of IFITM1, as previously reported (6,28). In addition to IFITM1, the expression of the related proteins IFITM2 and IFITM3 was examined, as they are often affected in KO studies due to their high sequence similarity. Western blot analysis confirmed that IFITM2 and IFITM3 expression was preserved in IFITM1 KO clones. However, a slight decrease in the protein levels is evident, particularly in the presence of IFNγ. This may be a consequence of the tight co-regulation within the IFITM family (6).

**Figure 2.**
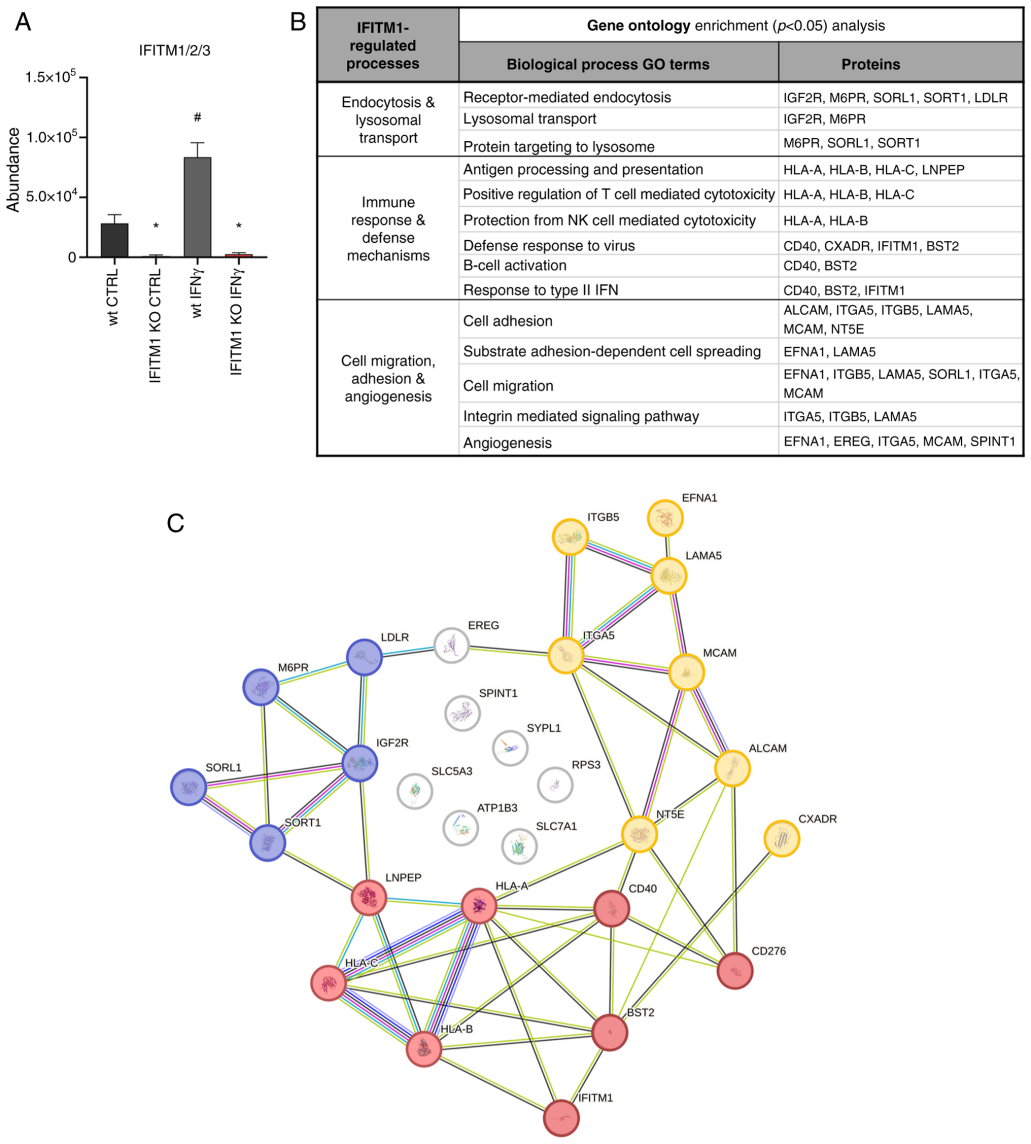
Functional bioinformatic analysis of IFITM1-regulated membrane proteins. (A) The abundance of common IFITM1/2/3 peptide was used as a measure of IFITM1 protein levels in SiHa wt and IFITM1 KO cells with and without IFNγ treatment. Error bars represent the means ± SEM from biological replicates. *P<0.05 when comparing wt and IFITM1 KO; ^#^P<0.05 when comparing untreated CTRL and respective IFNγ-treated samples. (B) Assignment of IFITM1-regulated proteins (fold change >1.5) to biological processes using DAVID Gene Ontology analysis (P<0.05). (C) Protein-protein interaction network of the significantly downregulated (fold change ≥1.5) proteins in IFITM1 KO cells (STRING database). Proteins are divided and color coded on the basis of their function: cell adhesion and migration (yellow); endocytosis, lysosomal transport and function (blue); and antigen presentation and immune response (red). IFITM, interferon-induced transmembrane protein; wt, wild-type; KO, knockout; CTRL, control.

A set of proteins that were significantly downregulated in IFITM1 KO cells with a fold change ≥1.5 and a significance level of P<0.05 were identified and quantified: 18 proteins without IFNγ treatment and 21 proteins after IFNγ treatment (Fig. 1C, Appendix S1). As expected, IFITM1 was the protein with the greatest difference in expression and was also induced by IFNγ (Fig. 2A). GO analysis using the DAVID bioinformatics tool (20) revealed that IFITM1-regulated proteins are involved primarily in receptor-mediated endocytosis, lysosomal transport of proteins, antiviral responses, antigen processing and presentation, regulation of immune system activity/function, and cell adhesion and migration (Fig. 2B). Using the STRING database of known and predicted protein-protein interactions (21), three major groups of interacting proteins that were significantly downregulated in IFITM1 KO cells were identified (Fig. 2C).

### IFITM1 protein regulates transcript abundance and modulates protein dynamics

**Figure 3.**
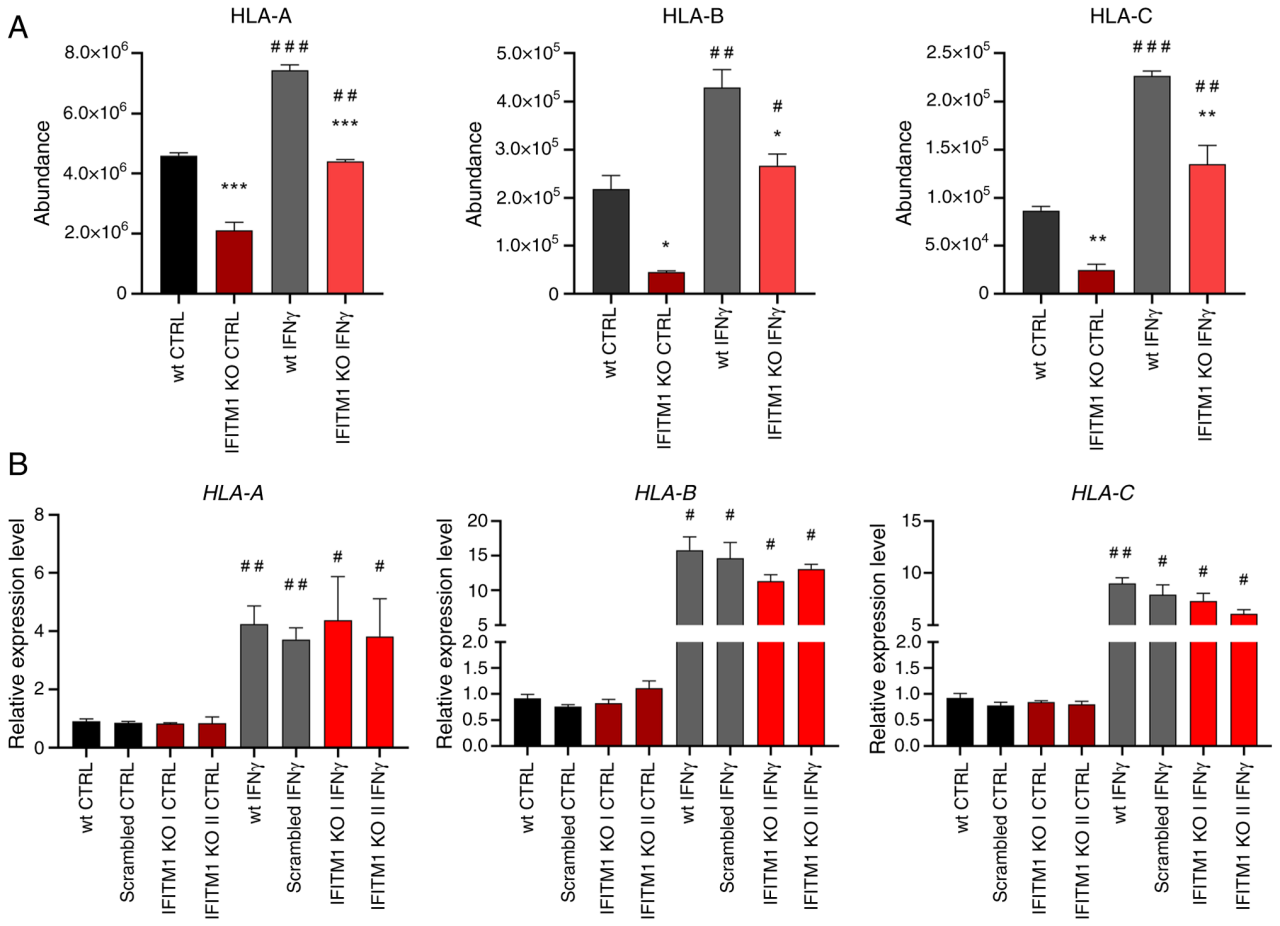
Membrane protein levels and associated gene expression of HLA class I molecules. (A) Peptide abundances determined by mass spectrometry analysis as a measure of HLA protein levels. (B) Determination of *HLA* gene expression by reverse transcription-quantitative PCR. *ACTB* was used as an endogenous control. The level of gene expression is relative to the value of the wt control (reference sample). The error bars represent the mean ± SEM from biological replicates. *P<0.05, **P<0.01, and ***P<0.001 when comparing wt and IFITM1 KO; ^#^P<0.05, ^##^P<0.01, and ^###^P<0.001 when comparing untreated CTRL and respective IFNγ-treated samples. HLA, human leukocyte antigen; wt, wild-type; KO, knockout; CTRL, control; IFN, interferon; IFITM, interferon-induced transmembrane protein.

The first group of proteins consists of proteins essential for antigen presentation and the immune response (Fig. 2C, red). IFITM1 KO cells presented reduced surface expression of all three major HLA class I molecules [HLA-A, HLA-B and HLA-C (Fig. 3A)] along with decreased levels of antigen-trimming leucyl and cystinyl aminopeptidase (LNPEP; Fig. S2). Although IFNγ treatment increased HLA abundance in both wt and IFITM1 KO cells, the levels remained significantly lower in IFITM1 KO cells. Gene expression analysis confirmed the increased transcriptional activity of *HLA* genes after IFNγ treatment in both cell lines, with no significant differences between them. These findings suggested that IFITM1 regulates HLA molecules at the post-translational level (Fig. 3B), which is consistent with the authors' previous findings concerning HLA-B in IFITM1/3 double-KO SiHa cells (29).

**Figure 4.**
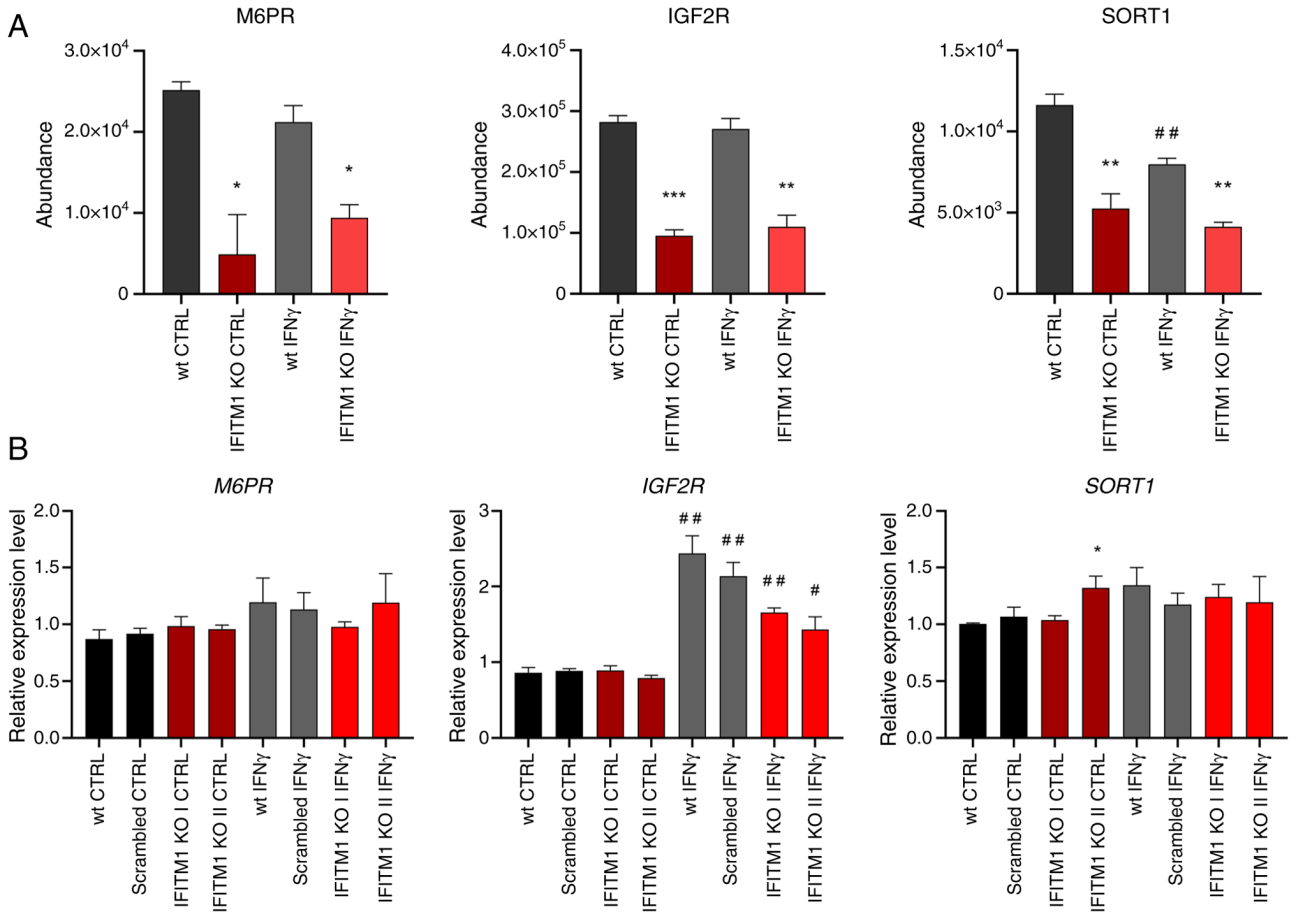
Membrane protein levels and associated gene expression in representatives of the functional group involved in endocytosis and lysosomal transport. (A) Peptide abundances determined by mass spectrometry analysis as a measure of the corresponding protein levels. (B) Determination of gene expression by reverse transcription-quantitative PCR. *ACTB* was used as an endogenous control. The level of gene expression is relative to the value of the wt control (reference sample). The error bars represent the mean ± SEM from biological replicates. *P<0.05, **P<0.01 and ***P<0.001 when comparing wt and IFITM1 KO; ^#^P<0.05 and ^##^P<0.01 when comparing untreated CTRL and respective IFNγ-treated samples. wt, wild-type; KO, knockout; CTRL, control; IFN, interferon; M6PR, mannose 6-phosphate receptor; SORT1, sortilin 1; IGF2R, insulin-like growth factor 2 receptor; IFITM, interferon-induced transmembrane protein.

The second group of proteins contains proteins pertaining to endocytosis, lysosomal transport, and lysosome function (Fig. 2C, blue), including mannose 6-phosphate (M6P) receptors (IGF2R and M6PR) and other M6P-independent sorting receptors [SORT1, sortilin related receptor 1 (SORL1) and LDLR] that are critical for protein trafficking from the Golgi apparatus to lysosomes and endosomes. IFNγ did not affect the plasma membrane levels of these proteins (Figs. 4A and S2). Interestingly, SORT1 levels were reduced upon IFNγ stimulation (Fig. 4A). Gene expression analysis of *M6PR* and *IGF2R* revealed no significant differences between wt and IFITM1 KO cells (Fig. 4B). Although surfaceome analysis revealed stable IGF2R membrane levels after IFNγ treatment, *IGF2R* gene expression was induced, which is consistent with reports in microglia (30). Conversely, *SORT1* gene expression remained unchanged under IFNγ treatment (Fig. 4B), highlighting cell-specific regulatory differences (31). Variability among IFITM1 KO clones was observed, with one clone displaying higher *SORT1* expression, which did not persist in the IFN-treated samples and was not reflected in the surfaceome analysis.

**Figure 5.**
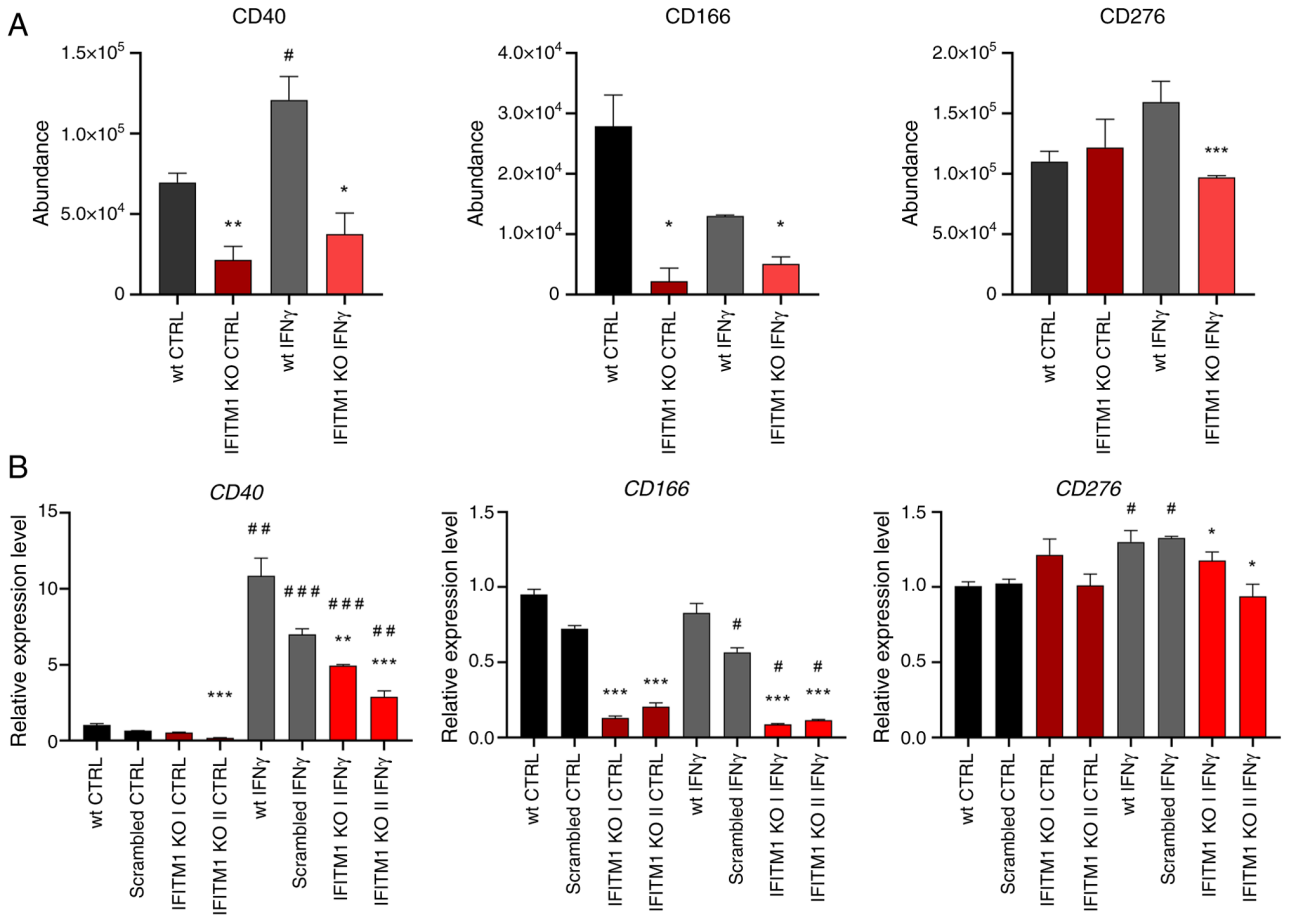
Membrane protein levels and associated gene expression in representatives of the functional group involved in cell adhesion and tumor-immune cell interactions. (A) Peptide abundances determined by mass spectrometry analysis as a measure of the corresponding protein levels. (B) Determination of gene expression by reverse transcription-quantitative PCR. *ACTB* was used as an endogenous control. The level of gene expression is relative to the value of the wt control (reference sample). The error bars represent the mean ± SEM from biological replicates. *P<0.05, **P<0.01 and ***P<0.001 when comparing wt and IFITM1 KO; ^#^P<0.05, ^##^P<0.01 and ^###^P<0.001 when comparing untreated CTRL and respective IFNγ-treated samples. wt, wild-type; KO, knockout; CTRL, control; IFN, interferon; IFITM, interferon-induced transmembrane protein.

The third group consists of cell adhesion molecules (CAMs) that mediate cell-cell and cell-extracellular matrix interactions (Fig. 2C, yellow). These multifunctional molecules often act as cell surface receptors, are overexpressed in cancer, and represent potential immunotherapy targets [for example, ALCAM-CD166; melanoma CAM (MCAM)-CD146/Mucin 18, CXADR] (32–34). Additionally, CD166, CD146 and CD40 play crucial roles in activating immune responses in tumor tissues. IFITM1-regulated CAMs include IFNγ-inducible (CD40, CD276) and non-IFNγ-inducible (CD166, MCAM, EFNA1, CXADR) proteins (Figs. 1C, 5A and S2). Changes in CD40 and CD166 membrane levels were found to be associated with their respective expression data (Fig. 5B), confirming that IFITM1 plays a significant role in regulating genes associated with tumor progression. Interestingly, IFITM1 depletion alone does not affect the expression or surface localization of CD276, an immune checkpoint molecule. However, upon IFNγ treatment, CD276 expression was upregulated at the RNA level in SiHa wt cells but not in IFITM1 KO cells. A similar trend was observed at the protein surface level; however, the induction of CD276 in SiHa wt cells did not reach statistical significance (P=0.06). These findings suggested that IFITM1 plays an important role in the regulation of CD276 by IFNγ and provide further evidence that IFITM1 acts as a master regulator not only for IRDS proteins but also for cell surface receptors.

### Functional analyses confirm the involvement of IFITM1 and IFITM3 in the mechanisms driving cancer progression

Following the analysis of the data obtained from MS, the effects of the IFITM1 protein were further validated by performing functional assays focused on cancer cell adhesion, migration, invasion and sphere formation-processes driven by changes in surfaceome composition. To verify the impact of the related IFITM3 protein and its possible relationship with IFITM1, independent IFITM3 KO and double-knockout IFITM1+3 KO SiHa derivatives, whose preparation has been previously described, were also tested (18).

**Figure 6.**
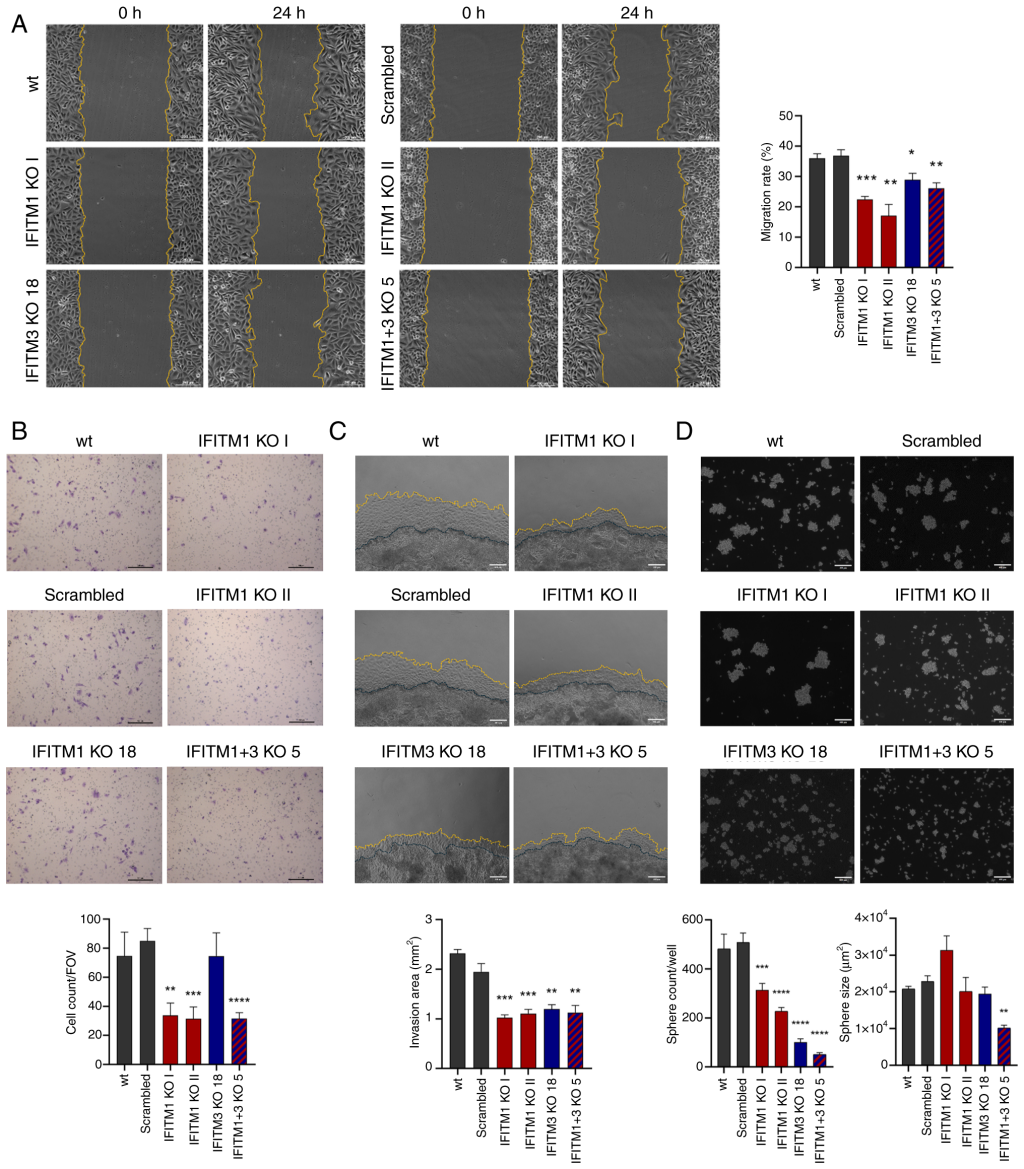
Evaluation of SiHa IFITM KO cell adhesion, migration and invasion abilities. (A) Representative images (magnification, ×100) of the wound healing (scratch) assay, with the borders of the scratch highlighted with yellow lines. The scratch area was determined at 0 and 24 h using Fiji ImageJ, and the values were used to calculate the migration rate (%). The average migration rates of IFITM1 KO, IFITM3 KO and IFITM1+3 KO cells in comparison with those of control cells (wt, scrambled) are shown in the graph. (B) Representative images of migratory cells after 24 h of Transwell assay (scale bar, 200 µm). The graph shows the average number of cells per field of view (FOV) for individual IFITM KO clones and their controls. (C) Representative images of cells invading (yellow line) from 3D Matrigel drops (black line) taken after 6 days (scale bar, 400 µm). The invasion area (mm^2^) was determined by Fiji ImageJ, and the average values for individual IFITM KO clones and controls are shown in the graph. (D) Representative images of 10-day cervicopheres formed by IFITM KO and control cells (scale bar, 400 µm). The average numbers of all spheres formed per well are compared in the left graph. The graph on the right shows the average sphere size (µm^2^) that was determined using Fiji ImageJ. The error bars represent the means from biological replicates ± SEM. *P<0.05, **P<0.01, ***P<0.001 and ****P<0.0001. IFITM, interferon-induced transmembrane protein; KO, knockout; wt, wild-type.

As expected, IFITM1 KO cells migrated more slowly compared with SiHa wt cells, as shown by the results of the wound healing (scratch) and Transwell migration assays, which were evaluated after 24 h of incubation (Fig. 6A and B). Interestingly, IFITM3 KO cells exhibited significantly reduced migration potential in the scratch assay, although the Transwell assay did not confirm this phenotype. When double-knockout IFITM1+3 KO cells were evaluated, a phenotype with low migratory activity was observed, similar to that of the IFITM1 KO clones.

To assess invasive ability, a 3D Matrigel drop assay was performed, which quantifies invading cells embedded in a Matrigel drop (26). This method confirmed a reduced capacity to invade through the Matrigel matrix in all tested IFITM KO clones, on the basis of the extent of the invasion area evaluated 6 days after Matrigel drop formation (Fig. 6C).

To evaluate the role of IFITM proteins in cell-cell adhesion and anchorage-independent growth, the ability of wt and IFITM1 KO and/or IFITM3 KO cells to form cervicospheres was compared. All the tested IFITM KO clones exhibited a significantly reduced capacity to grow as spheres, suggesting that the IFITM1 and IFITM3 proteins influence intercellular interactions and cell-extracellular matrix adhesion (Fig. 6D). Notably, compared with IFITM1 KO cells, IFITM3 KO cells displayed a greater reduction in sphere formation. However, the most pronounced reduction in both the number and size of spheres was observed in IFITM1+3 KO cells.

### IFITM1 and IFITM3 KO cells show reduced expression of immunity-related CD markers and increased resistance to NK cell killing

Given the identified group of IFITM1-regulated proteins involved in immune responses, two selected proteins were further validated, CD40 and CD166, which are critical factors in tumor-immune cell interactions.

**Figure 7.**
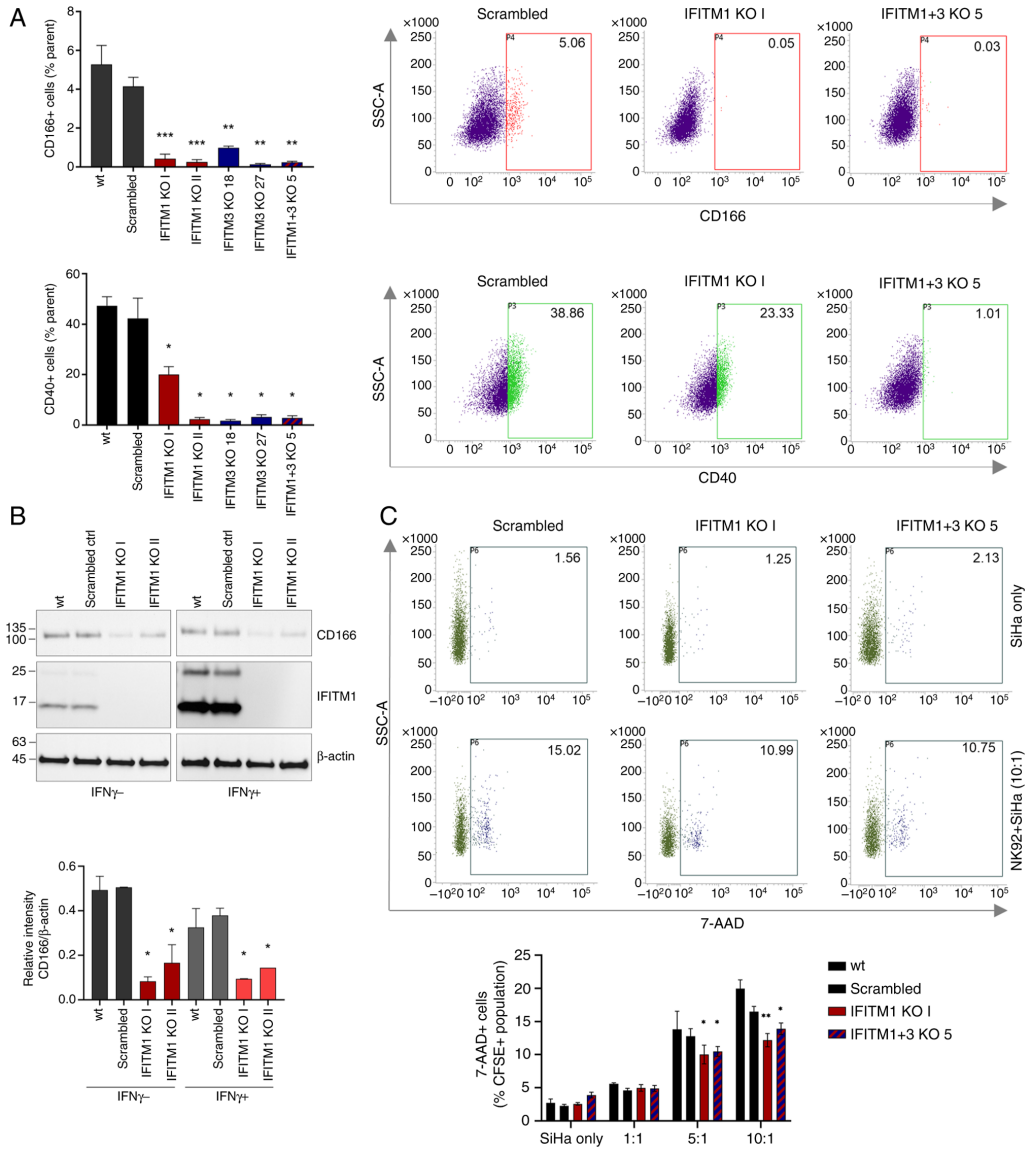
Evaluation of immune-related CD166 and CD40 proteins in the IFITM KO background. (A) FACS analysis of CD166 (upper panel) and CD40 (bottom panel) cell surface levels in SiHa IFITM1/3 KO clones and controls (wt, scrambled). The graphs display the mean percentage of positive cells within the parental populations, as shown in dot plots of selected representative samples. (B) Total protein levels of CD166 and IFITM1 in IFITM1 KO SiHa cells with or without IFNγ stimulation. β-actin served as loading control. The signal intensity relative to that of β-actin was calculated using Fiji ImageJ. (C) FACS analysis of dead SiHa IFITM1/3 KO cells and controls (7-AAD^+^ within the CFSE^+^ population) after 4 h of co-culture with NK cells. Representative dot plots show the percentage of dead SiHa cells cultured alone (upper panels) or co-cultured with NK92 cells at a 10:1 ratio (NK:SiHa, bottom panels). The graph presents the mean percentage of 7-AAD^+^ SiHa cells co-cultured with NK92 cells at the indicated ratios. The error bars indicate the means ± SEM from biological replicates. *P<0.05, **P<0.01 and ***P<0.001. IFITM, interferon-induced transmembrane protein; KO, knockout; wt, wild-type; IFN, interferon; NK, natural killer.

FACS analysis confirmed reduced CD40 and CD166 levels in all the tested IFITM KO cell clones (Figs. 7A and S3A). Immunoblotting also revealed a decreased CD166 expression at the total protein level (Fig. 7B). Consistent with the MS and qPCR findings, immunoblotting of SiHa cell lysates revealed that CD166 expression was not responsive to IFNγ stimulation (Fig. 7B).

Using the NK cytotoxicity assay, which involves the co-cultivation of tumor and NK cells followed by an analysis of tumor cell viability, the influence of IFITM proteins on intercellular communication and the ability of NK cells to recognize and eliminate tumor cells were examined. This assay demonstrated that both IFITM1 KO and IFITM1+3 KO cells are significantly more resistant to NK cell-mediated killing, as evidenced by a lower percentage of dead (7-AAD^+^) tumor cells after 4 h of co-cultivation (Figs. 7C and S3B).

## Discussion

Despite advancements in prevention, detection and treatment, cervical cancer remains a leading cause of cancer-related mortality in women. Treatment is stage-dependent (TNM, International Federation of Gynecology and Obstetrics classification), with advanced cases requiring a combination of surgery, radiotherapy, chemotherapy, or targeted therapy (2). Cisplatin remains the cornerstone of chemotherapy, used alone or in combination, including as neoadjuvant therapy to reduce tumor mass before surgery (35). Significant progress has been made in targeted therapies, with bevacizumab (anti-vascular endothelial growth factor) and pembrolizumab (anti-PD-1 immunotherapy) approved for recurrent and metastatic cervical cancer (36,37). Ongoing clinical trials are investigating additional approaches, such as poly (ADP-ribose) polymerase inhibitors, mammalian target of rapamycin inhibitors and therapeutic HPV vaccines (38).

Despite these advances, the prognosis for advanced-stage disease remains poor due to tumor heterogeneity and therapy resistance. The shift toward precision medicine underscores the need for prognostic and predictive biomarkers to improve patient stratification and treatment outcomes (3,4). In this context, surfaceome analysis represents a powerful tool for identifying therapeutic targets by profiling cell surface proteins.

IFITM1 was originally identified as a leukocyte membrane surface antigen and is now known primarily for its significant antiviral activity (39). Over the past decade, increasing evidence has shown that IFITM1 is overexpressed in numerous human solid tumors (6,40). Aberrant expression of IFITM1 promotes tumor cell proliferation, inhibits cell death, stimulates invasion and metastasis, contributes to cancer cell resistance to endocrine therapy, chemotherapy and radiotherapy, and has prognostic value for patient outcomes (6,11,40). IFITM1 is also part of a subset of IFN-responsive genes that contribute to the IRDS signature. The authors have demonstrated that the suppression of IFITM1 via siRNA targeting can impair the IRDS network (18,41). Current knowledge indicates that IFITM proteins affect membrane structure and function, intracellular trafficking, and the turnover of cell surface receptors (15). IFITM1 has also been reported to be a tight junction protein (42,43). It was previously demonstrated that knocking out the IFITM1 and IFITM3 proteins led to the downregulation of HLA-B expression on the surface of cervical cancer cells, suggesting an explanation for the inverse correlation between IFITM1/3 expression and metastasis formation (18). In the present study, it was confirmed that, in addition to HLA-B, IFITM1 regulates a whole set of surface proteins in cervical cancer cells, both with and without dependence on IFNγ. Furthermore, IFITM1 knockout affects the regulation of specific protein surface expression, either at the level of membrane dynamics or gene transcription.

Most published studies focus on either one IFITM family member or the combined effect of several IFITMs without distinguishing individual members. This is because of the high homology between IFITM proteins (6), which makes specific targeting difficult. However, recent studies have overcome these obstacles and are now describing the different functions of IFITM proteins (44–47). This phenomenon is particularly intriguing among such homologous proteins. Therefore, in addition to focusing on IFITM1, validation and functional assays were enriched with IFITM3 KO cells, as well as ‘double KO’ cells depleted of both the IFITM1 and IFITM3 proteins, by the CRISPR/Cas9 gene-editing method to reveal their synergistic effects.

SILAC-based quantitative MS is a widely used proteomic approach that enables the quantification and evaluation of protein dynamics within living cells. An additional step involving surface-protein biotinylation allowed us to enrich samples with surface/membrane proteins, whose detection in total lysates is challenging owing to their hydrophobic nature and the abundance of intracellular proteins. Given the location of IFITM1 in the plasma membrane and its known role in altering membrane properties during antiviral responses, the surfaceomes of IFITM1 KO cells and parental SiHa wt cells were measured with or without IFNγ treatment. The SILAC approach allowed us to evaluate the changes in protein levels induced by the treatment and compare the membrane protein levels between IFITM1 KO and SiHa wt cells, including effects that were dependent on or independent of the presence of IFNγ.

A total of 28 proteins that were differentially expressed in wt and IFITM1 KO cells with a fold change of ≥1.5. Among these proteins, 9 were significantly stimulated by IFNγ. The present results suggested that IFITM1 affects surfaceome composition even without IFNγ stimulation, at least in SiHa cell line, which has a relatively high basal level of IFITM1. Furthermore, the changes upon IFNγ stimulation indicate the possible involvement of IFITM1 in the IFN-dependent regulation of differentially expressed proteins or possible changes in the functional properties of IFITM1 itself in the presence of IFN. A detailed understanding of how IFITM1 contributes to IFN signaling and the subsequent expression of IFN-inducible proteins deserves further study.

As expected, the protein with the greatest difference in expression between wt and IFITM1 KO cells was IFITM1. Only one IFITM peptide was detected by MS, and this peptide is common to the IFITM1, IFITM2 and IFITM3 proteins, thus distinguishing between them was not possible. Western blot analysis confirmed that IFITM1 expression was specifically ablated in IFITM1 KO cells, while IFITM2 and IFITM3 expression was preserved, albeit with a slight decrease, particularly in the presence of IFNγ. This reduction may result from the tight co-regulation of these proteins due to shared regulatory elements, the stabilizing effects of hetero-oligomer formation within cellular membranes, and alterations in IFN response dynamics caused by IFITM1 KO, which may further influence the expression of IFN-stimulated genes, including *IFITM*s, through feedback regulation (6,18). Together with the predominant localization of IFITM1 on the cell surface (in contrast to IFITM2 and IFITM3, which are mostly located in endosomes/lysosomes), it was hypothesized that the identified peptide originated from IFITM1, with a minor contribution from IFITM2/3, which can be temporarily found on the cell surface before their internalization into endosomes (48). This also explains the presence of small amounts of the IFITM peptide in the IFITM1 KO samples.

Despite the advantages of quantitative proteomics, limitations in peptide quantification and reproducibility may lead to the exclusion of relevant targets. Nonetheless, this approach enabled us to identify entire functional protein groups, which led to the discovery of IFITM1-regulated processes central to our study. The proteins, whose levels in the plasma membrane were downregulated by IFITM1 knockout, can be classified into three main groups according to their function: Endocytosis and lysosomal transport, antigen processing and presentation together with regulation of the immune response, and cell adhesion and migration. The regulation of the rate and manner of endocytosis controls the exposure and abundance of membrane proteins at the cell surface. Once internalized, proteins undergo recycling or degradation in lysosomes through autophagy. IFITM proteins are known to modulate membrane rigidity and curvature, cholesterol content, trafficking of endocytosed cargo, and endosomal/lysosomal pH, thereby affecting viral entry into cells (12,13,15–17,49). According to our MS data, IFITM1 affects several proteins linked to receptor-mediated endocytosis and endosomal/lysosomal trafficking (LDLR, IGF2R, M6PR, SORT1 and SORL1). On the basis of the unchanged gene expression levels of selected representatives of this functional group (IGF2R, M6PR and SORT1) in IFITM1 KO cells, it was hypothesized that, along with effects on the biophysical properties of membranes, IFITM1 affects the exposure and turnover of numerous membrane proteins, which may be another explanation for its broad antiviral properties and underscores its significant role in tumor progression. A more detailed description of how IFITM proteins regulate the stability and localization of other proteins is the subject of our further investigation.

The dysregulation of cell migration, adhesion and invasion regulators, such as MCAM/CD146, ALCAM/CD166, CAR/CXADR, EFNA1, LAMA5, CD73/NT5E and integrins, contributes to cancer progression and metastasis (50–55). The importance of IFITM1 in these processes is supported by functional assays showing a decreased ability of IFITM1 KO cells to migrate (toward the scratch or across the porous membrane) and invade through Matrigel, which represents the extracellular matrix. These results are consistent with findings in various tumor types reported in the literature (56–58). However, little is known about IFITM proteins in cervical cancer. In contrast, Zheng *et al* (59) reported that migration was inhibited after the transfection of HeLa cells with an IFITM1 recombinant construct. While IFITM1 KO clones showed consistent results across migration assays, the IFITM3 KO 18 clone exhibited a discrepancy. IFITM3 KO cells migrated similarly to controls in the Transwell assay, but their migratory ability was significantly reduced in the scratch assay. This may be due to the distinct principles of each assay: the Transwell assay measures individual cell migration, where cytoskeletal components are crucial, while the scratch assay focuses on collective migration, which relies on cell-cell adhesion molecules. Both IFITM1 and IFITM3 are associated with membrane structure and dynamics, which are closely linked to cytoskeletal remodeling. However, their functions may differ due to differences in their localization and expression patterns (6).

To evaluate adhesion properties, the cells were allowed to proliferate in low-attachment plates, forcing them to adhere to each other and form cervicospheres. IFITM1 and IFITM3 KO clones showed a diminished ability to form spheres, as evaluated on the basis of sphere counts. Compared with single KO, the knockout of both IFITM1 and IFITM3 had a synergistic effect. Interestingly, when evaluating the size of the formed spheres, clones deficient in one of the IFITM proteins did not differ from the controls, whereas deficiency in both proteins led to significantly smaller cell aggregates. Sphere formation assays are typically used to evaluate stem cell properties. However, changes in stem cell markers among IFITM KO clones (data not shown) were not observed, indicating that the observed differences are due to alterations in adhesion proteins.

Surfaceome analysis confirmed our previous findings that IFITM proteins affect the exposure of MHC-I molecules at the surface of cervical cancer cells (18), which might be important for cancer cell interactions with the immune system, supporting tumor immune escape (60). These findings were recently confirmed by She *et al* (61), who described the effect of IFITM1 expression on the brain colonization of human lung cancer cells through the regulation of the membrane localization of MHC-I and the enhancement of CD8^+^ T-cell cytolytic activity. Interestingly, it was also shown that IFITM1 deficiency does not affect the expression level of *HLA* genes, suggesting that IFITM1 instead affects the dynamics of proteins within the membrane. This finding is consistent with our previous results on the *HLA-B* gene (29). Additionally, IFN-independent LNPEP aminopeptidase, which is important for antigen trimming before MHC-I-mediated presentation (62), was identified among the downregulated molecules in IFITM1 KO cells. Other proteins regulated by IFITM1 that could be involved in antitumor immune responses include CD40 (63–65), CD166 (32,66), MCAM/CD146 (67,68) and CXADR (52). Interestingly, differences were observed in the involvement of the IFNγ signaling pathway in the regulation of CD markers. The membrane levels of the IFN-inducible CD40 and CD276 proteins correlated with the gene expression levels. IFITM1 knockout leads to CD40 downregulation irrespective of the presence of IFNγ and abolishes IFN-induced CD276 expression, which is also reflected in the membrane exposure of the protein. Furthermore, IFITM1 decreases the expression of CD166, which is unresponsive to IFNγ treatment, as confirmed by SILAC, qPCR and western blot analysis. The surface levels of the CD40 and CD166 proteins were further validated and significant downregulation of the IFITM1 and IFITM3 KO SiHa derivatives was confirmed. Consistent with the differential levels of immune-related proteins, IFITM1 KO and IFITM1+3 KO clones escaped the effects of NK cells, as demonstrated by the NK cytotoxicity assay. Other studies that link IFITM proteins with innate and adaptive immunity are emerging, highlighting the potential role of these proteins in tumor immunosurveillance (69–71). Uncovering the exact mechanism by which IFITMs regulate these proteins and thereby hinder antitumor immunity will be the subject of our further investigations.

In conclusion, the importance of the IFITM1 and IFITM3 proteins in processes that depend on the composition of proteins in the plasma membrane, such as cell adhesion, migration, invasion and interaction with immune cells was confirmed. The presented results confirm the authors' hypothesis that IFITM proteins regulate the expression and membrane exposure of proteins, thereby facilitating tumor progression while also supporting tumor immunosurveillance. The precise mechanism by which IFITM1 modulates the membrane presentation of identified proteins remains the subject of ongoing research.

## Supplementary Material

Supporting Data

Supporting Data

## Data Availability

The data generated in the present study may be requested from the corresponding author. The data generated in the present study may be found in the PRIDE database under accession number PXD053951 or at the following URL: https://www.ebi.ac.uk/pride/archive/projects/PXD053951.

## Abbreviations

7-AAD: 7-aminoactinomycin D
ALCAM: activated leukocyte cell adhesion molecule
CAM: cell adhesion molecules
CFSE: carboxy-fluorescein succinimidyl ester
CXADR: coxsackie virus and adenovirus receptor
ECL: enhanced chemiluminescence
EFNA1: ephrin A1
FASP: filter-aided sample preparation protocol
FDR: false discovery rate
GO: Gene Ontology
HLA: human leukocyte antigen
IFITM: interferon-induced transmembrane protein
IFN: interferon
IGF2R: insulin-like growth factor 2 receptor
IRDS: IFN-related DNA damage resistance signature
KO: knockout
LDLR: low-density lipoprotein receptor
LNPEP: leucyl and cystinyl aminopeptidase
M6P: mannose-6-phosphate
M6PR: M6P receptor
MCAM: melanoma CAM
MHC-I: major histocompatibility complex class I
PBST: phosphate-buffered saline with Tween 20
RSLC: rapid separation liquid chromatography
SILAC: stable isotope labeling with amino acids in cell culture
SORL1: sortilin related receptor 1
SORT1: sortilin 1
