# Supplementary material for: IFITM1 as a modulator of surfaceome dynamics and aggressive phenotype in cervical cancer cells
Source: Oncol Rep. 2025 Apr 29;53(6):71. doi: 10.3892/or.2025.8904 (PMC12059461; doi:10.3892/or.2025.8904)
Supplement: Supporting Data [file Supplementary_Data1.pdf]

Appendix S1. MS data for IFITM1 KO and wt SiHa cells, with or without IFNγ treatment. Individual comparisons are listed in separate tables: wt ctrl x IFITM1 KO ctrl, wt IFNγ x IFITM1 KO IFNγ, wt IFNγ x wt ctrl, IFITM1 KO IFNγ x IFITM1 KO ctrl. The identified proteins are sorted in descending order according to the protein abundance ratio values. Proteins of non-human origin have been removed as contaminants. The p-value was determined using an unpaired t-test on the measurement results from the biological triplicates. In cases where fewer than two values were measured in a given biological triplicate, the p-value was not determined and was marked as "not quantified" (nq).

Accession	Description	Gene symbol	Abundance Ratio: (WT, ctrl) / (KO IFITM1, ctrl)	P-value
P13164	Interferon-induced transmembrane protein 1	IFITM1	6,938	nq
P30486	HLA class I histocompatibility antigen, B-48 alpha chain	HLA-B	4,53	0,004
P25942	Tumor necrosis factor receptor superfamily member 5	CD40	4,064	0,009
P30479	HLA class I histocompatibility antigen, B-41 alpha chain	HLA-B	3,113	0,109
P20827	Ephrin-A1	EFNA1	2,951	0,006
Q13740	CD166 antigen	ALCAM	2,889	nq
B7Z368	Uncharacterized protein C10orf142	LOC100130539; C10orf142	2,72	0,084
O14944	Proepiregulin	EREG	2,663	0,096
P20645	Cation-dependent mannose-6-phosphate receptor	M6PR	2,583	nq
Q99523	Sortilin	SORT1	2,403	0,005
P11279	Lysosome-associated membrane glycoprotein 1	LAMP1	2,367	0,260
Q9UIQ6	Leucyl-cystinyl aminopeptidase	LNPEP	2,349	0,044
P55290	Cadherin-13	CDH13	2,347	0,062
Q9NZJ5	Eukaryotic translation initiation factor 2-alpha kinase 3	EIF2AK3	2,249	0,299
P01130	Low-density lipoprotein receptor	LDLR	2,246	0,002
P43121	Cell surface glycoprotein MUC18	MCAM	2,149	0,029
P11717	Cation-independent mannose-6-phosphate receptor	IGF2R	2,144	0,000
P15941	Mucin-1	MUC1	2,108	nq
P04222	HLA class I histocompatibility antigen, Cw-3 alpha chain	HLA-C	2,102	0,003

P53794	sodium/myo-inositol cotransporter	SLC5A3	2,093	0,001
P05534	HLA class I histocompatibility antigen, A-24 alpha chain	HLA-A	2,093	0,001
P30825	High affinity cationic amino acid transporter 1	SLC7A1	2,001	0,011
P0C854	Putative cat eye syndrome critical region protein 9	CECR9	1,88	0,347
Q07954	prolow-density lipoprotein receptor-related protein 1	LRP1	1,845	0,442
P49184	Deoxyribonuclease-1-like 1	DNASE1L1	1,814	0,113
Q92673	Sortilin-related receptor	SORL1	1,811	0,003
Q10589	bone marrow stromal antigen 2	BST2	1,809	0,011
Q9NPH3	Interleukin-1 receptor accessory protein	IL1RAP	1,792	0,700
Q13443	disintegrin and metalloproteinase domain-containing protein 9	ADAM9	1,773	0,058
P29323	Ephrin type-B receptor 2	EPHB2	1,751	0,175
P09603	Macrophage colony-stimulating factor 1	CSF1	1,726	0,152
P25445	Tumor necrosis factor receptor superfamily member 6	FAS	1,712	nq
Q6PL18	ATPase family AAA domain-containing protein 2	ATAD2	1,691	0,286
Q6P4Q7	metal transporter CNNM4	CNNM4	1,662	0,020
P18084	Integrin beta-5	ITGB5	1,662	0,468
Q8NFJ5	Retinoic acid-induced protein 3	GPRC5A	1,62	0,161
Q16563	Synaptophysin-like protein 1	SYPL1	1,577	0,016
P15328	Folate receptor alpha	FOLR1	1,567	nq
P61769	Beta-2-microglobulin	B2M	1,519	0,072
P42892	Endothelin-converting enzyme 1	ECE1	1,513	0,305
P54709	sodium/potassium-transporting ATPase subunit beta-3	ATP1B3	1,499	0,000
P78310	Coxsackievirus and adenovirus receptor	CXADR	1,496	0,024
P22695	Cytochrome b-c1 complex subunit 2, mitochondrial	UQCRC2	1,496	0,056
P54760	Ephrin type-B receptor 4	EPHB4	1,491	0,519
Q969P0	Immunoglobulin superfamily member 8	IGSF8	1,485	0,004

Q96NT5	Proton-coupled folate transporter	SLC46A1	1,474	0,220
Q13308	Inactive tyrosine-protein kinase 7	PTK7	1,47	0,300
P02786	Transferrin receptor protein 1	TFRC	1,469	0,285
Q8IWT6	volume-regulated anion channel subunit LRRC8A	LRRC8A	1,468	0,198
P60033	CD81 antigen	CD81	1,467	0,017
Q15262	Receptor-type tyrosine-protein phosphatase kappa	PTPRK	1,452	0,001
Q15043	Zinc transporter ZIP14	SLC39A14	1,445	0,161
Q687X5	Metalloreductase STEAP4	STEAP4	1,422	0,142
P06756	Integrin alpha-V	ITGAV	1,4	0,010
Q9NUM4	Transmembrane protein 106B	TMEM106B	1,398	0,604
P08648	Integrin alpha-5	ITGA5	1,396	0,059
Q92542	Nicastrin	NCSTN	1,393	0,015
Q3ZCN5	Otogelin-like protein	OTOGL	1,378	0,347
Q96QD8	sodium-coupled neutral amino acid transporter 2	SLC38A2	1,354	0,913
Q9BZM5	UL16-binding protein 2	ULBP2	1,345	0,089
Q9Y289	Sodium-dependent multivitamin transporter	SLC5A6	1,342	0,168
P05026	Sodium/potassium-transporting ATPase subunit beta-1	ATP1B1	1,341	0,007
Q04941	proteolipid protein 2	PLP2	1,339	0,329
P48960	CD97 antigen	CD97; ADGRE5	1,336	0,222
O15427	Monocarboxylate transporter 4	SLC16A3	1,311	0,308
A5PL33	protein KRBA1	KRBA1	1,291	0,689
P14384	Carboxypeptidase M	CPM	1,28	0,113
P18827	syndecan-1	SDC1	1,277	0,160
Q08722	Leukocyte surface antigen CD47	CD47	1,273	0,406
P41440	Folate transporter 1	SLC19A1	1,272	0,324
P04843	Dolichyl-diphosphooligosaccharide--protein glycosyltransferase subunit 1	RPN1	1,261	0,631

Q29983	MHC class I polypeptide-related sequence A	MICA	1,259	0,041
Q04912	Macrophage-stimulating protein receptor	MST1R	1,258	0,280
Q9H5V8	CUB domain-containing protein 1	CDCP1	1,25	0,577
Q8WVN6	Secreted and transmembrane protein 1	SECTM1	1,246	nq
Q6NSJ0	Uncharacterized family 31 glucosidase KIAA1161	KIAA1161; MYORG	1,242	0,551
Q15417	Calponin-3	CNN3	1,239	0,605
Q99714	3-hydroxyacyl-CoA dehydrogenase type-2	HSD17B10	1,235	nq
P04626	Receptor tyrosine-protein kinase erbB-2	ERBB2	1,221	0,113
P17813	Endoglin	ENG	1,216	0,336
Q9P2B2	prostaglandin F2 receptor negative regulator	PTGFRN	1,215	0,690
P35052	Glypican-1	GPC1	1,212	0,063
P04920	Anion exchange protein 2	SLC4A2	1,209	0,559
P09758	Tumor-associated calcium signal transducer 2	TACSTD2	1,203	0,650
Q99650	Oncostatin-M-specific receptor subunit beta	OSMR	1,201	0,263
P48509	CD151 antigen	CD151	1,2	0,136
P15529	Membrane cofactor protein	CD46	1,199	0,764
Q6YHK3	CD109 antigen	CD109	1,199	0,000
Q9UNN8	Endothelial protein C receptor	PROCR	1,199	0,108
P35613	Basigin	BSG	1,183	0,114
P27824	Calnexin	CANX	1,183	0,666
P05023	Sodium/potassium-transporting ATPase subunit alpha-1	ATP1A1	1,182	0,150
P53985	Monocarboxylate transporter 1	SLC16A1	1,18	0,059
O15431	High affinity copper uptake protein 1	SLC31A1	1,171	0,125
P08174	Complement decay-accelerating factor	CD55	1,163	0,488
O15031	Plexin-B2	PLXNB2	1,158	0,044
P09382	Galectin-1	LGALS1	1,156	0,674

Q12913	Receptor-type tyrosine-protein phosphatase eta	PTPRJ	1,153	0,568
P33527	Multidrug resistance-associated protein 1	ABCC1	1,152	0,109
Q13641	Trophoblast glycoprotein	TPBG	1,148	0,594
P19256	Lymphocyte function-associated antigen 3	CD58	1,146	0,606
Q8IWA5	Choline transporter-like protein 2	SLC44A2	1,138	0,105
Q13421	Mesothelin	MSLN	1,127	0,622
Q9H2H9	Sodium-coupled neutral amino acid transporter 1	SLC38A1	1,125	0,676
P43007	neutral amino acid transporter A	SLC1A4	1,124	0,367
P13726	tissue factor	F3	1,115	0,958
Q14126	Desmoglein-2	DSG2	1,112	0,861
Q03167	Transforming growth factor beta receptor type 3	TGFBR3	1,112	0,447
P26006	Integrin alpha-3	ITGA3	1,112	0,858
P15529	Membrane cofactor protein	CD46	1,111	0,510
P15151	Poliovirus receptor	PVR	1,106	0,664
P78324	Tyrosine-protein phosphatase non-receptor type substrate 1	SIRPA	1,103	0,581
Q9H6X2	Anthrax toxin receptor 1	ANTXR1	1,101	0,687
Q9NV96	Cell cycle control protein 50A	TMEM30A	1,096	0,209
Q92692	Nectin-2	PVRL2; NECTIN2	1,08	0,061
Q15758	Neutral amino acid transporter B(0)	SLC1A5	1,079	0,298
P16070	CD44 antigen	CD44	1,068	0,799
P05362	Intercellular adhesion molecule 1	ICAM1	1,059	0,643
Q8NE01	Metal transporter CNNM3	CNNM3	1,056	0,868
Q9ULI3	Protein HEG homolog 1	HEG1	1,054	0,662
P23396	40S ribosomal protein S3	RPS3	1,04	0,952
Q4KMQ2	Anoctamin-6	ANO6	1,038	0,383
Q13332	Receptor-type tyrosine-protein phosphatase S	PTPRS	1,021	0,321

Q15084	Protein disulfide-isomerase A6	PDIA6	1,018	0,526
P11586	C-1-tetrahydrofolate synthase, cytoplasmic	MTHFD1	1,011	nq
P50281	Matrix metalloproteinase-14	MMP14	0,997	0,806
Q9Y6M5	zinc transporter 1	SLC30A1	0,995	0,985
P21926	CD9 antigen	CD9	0,994	0,649
P30530	Tyrosine-protein kinase receptor UFO	AXL	0,991	0,680
Q9P035	Very-long-chain (3R)-3-hydroxyacyl-CoA dehydratase 3	PTPLAD1; HACD3	0,984	0,762
O75144	ICOS ligand	ICOSLG; LOC102723996	0,982	0,871
P11166	Solute carrier family 2, facilitated glucose transporter member 1	SLC2A1	0,978	0,844
Q5ZPR3	CD276 antigen	CD276	0,96	0,662
P08195	4F2 cell-surface antigen heavy chain	SLC3A2	0,954	0,680
P08195	4F2 cell-surface antigen heavy chain	SLC3A2	0,954	0,922
Q9UIW2	Plexin-A1	PLXNA1	0,953	0,371
O43278	Kunitz-type protease inhibitor 1	SPINT1	0,95	0,749
O15230	Laminin subunit alpha-5	LAMA5	0,945	0,877
P98172	ephrin-B1	EFNB1	0,942	0,374
P08069	Insulin-like growth factor 1 receptor	IGF1R	0,938	0,970
P08729	Keratin, type II cytoskeletal 7	KRT7	0,931	0,889
Q96J84	Kin of IRRE-like protein 1	KIRREL; KIRREL1	0,929	0,172
Q92859	neogenin	NEO1	0,922	0,640
P31641	Sodium- and chloride-dependent taurine transporter	SLC6A6	0,922	0,790
Q7L1W4	volume-regulated anion channel subunit LRRC8D	LRRC8D	0,92	0,113
P42224	Signal transducer and activator of transcription 1-alpha/beta	STAT1	0,918	0,644
P51149	ras-related protein Rab-7a	RAB7A	0,917	0,659
P23634	Plasma membrane calcium-transporting ATPase 4	ATP2B4	0,916	0,840
O95297	Myelin protein zero-like protein 1	MPZL1	0,911	0,075

Q92973	transportin-1	TNPO1	0,9	0,406
P13987	CD59 glycoprotein	CD59	0,896	0,844
P10586	Receptor-type tyrosine-protein phosphatase F	PTPRF	0,881	0,211
P50895	Basal cell adhesion molecule	BCAM	0,878	0,684
Q15388	Mitochondrial import receptor subunit TOM20 homolog	TOMM20	0,874	0,809
P14209	CD99 antigen	CD99	0,871	0,848
P14923	Junction plakoglobin	JUP	0,868	0,738
Q14118	Dystroglycan	DAG1	0,865	0,844
Q12907	Vesicular integral-membrane protein VIP36	LMAN2	0,86	0,727
P05556	Integrin beta-1	ITGB1	0,854	0,645
Q5RHP9	glutamate-rich protein 3	C1orf173; ERICH3	0,854	0,558
P13473	Lysosome-associated membrane glycoprotein 2	LAMP2	0,848	nq
P00533	epidermal growth factor receptor	EGFR	0,846	0,336
Q8TCZ2	CD99 antigen-like protein 2	CD99L2	0,833	0,726
P50454	Serpin H1	SERPINH1	0,83	0,573
P62979	Ubiquitin-40S ribosomal protein S27a	RPS27A	0,829	0,639
Q8WTV0	Scavenger receptor class B member 1	SCARB1	0,826	0,656
Q8WWI5	choline transporter-like protein 1	SLC44A1	0,824	nq
P32004	Neural cell adhesion molecule L1	L1CAM	0,821	0,314
P62937	peptidyl-prolyl cis-trans isomerase A	PPIA	0,817	nq
O14672	Disintegrin and metalloproteinase domain-containing protein 10	ADAM10	0,813	0,311
P68363	Tubulin alpha-1B chain	TUBA1B	0,812	0,268
Q03405	Urokinase plasminogen activator surface receptor	PLAUR	0,811	0,319
Q01650	large neutral amino acids transporter small subunit 1	SLC7A5	0,81	0,156
P08962	CD63 antigen	CD63	0,809	0,591
Q86V24	Adiponectin receptor protein 2	ADIPOR2	0,807	0,922

O00592	Podocalyxin	PODXL	0,805	0,368
P05187	alkaline phosphatase, placental type	ALPP	0,804	nq
P78380	Oxidized low-density lipoprotein receptor 1	OLR1	0,804	0,040
P07437	tubulin beta chain	TUBB	0,798	0,173
P63000	Ras-related C3 botulinum toxin substrate 1	RAC1	0,797	0,516
O00571	ATP-dependent RNA helicase DDX3X	DDX3X	0,791	0,044
Q04695	Keratin, type I cytoskeletal 17	KRT17	0,786	0,466
O75153	Clustered mitochondria protein homolog	CLUH; KIAA0664	0,785	nq
P04844	Dolichyl-diphosphooligosaccharide--protein glycosyltransferase subunit 2	RPN2	0,779	0,544
P17301	Integrin alpha-2	ITGA2	0,778	nq
P16615	Sarcoplasmic/endoplasmic reticulum calcium ATPase 2	ATP2A2	0,765	0,824
P31431	syndecan-4	SDC4	0,759	0,130
P21796	voltage-dependent anion-selective channel protein 1	VDAC1	0,759	0,162
P43307	Translocon-associated protein subunit alpha	SSR1	0,754	0,321
P23284	peptidyl-prolyl cis-trans isomerase B	PPIB	0,752	0,552
Q00341	Vigilin	HDLBP	0,752	0,185
P06576	ATP synthase subunit beta, mitochondrial	ATP5B	0,75	nq
Q9Y639	Neuroplastin	NPTN	0,75	0,033
Q04721	Neurogenic locus notch homolog protein 2	NOTCH2	0,746	0,581
P31327	Carbamoyl-phosphate synthase [ammonia], mitochondrial	CPS1	0,743	0,185
P09622	Dihydrolipoyl dehydrogenase, mitochondrial	DLD	0,74	0,233
O14763	Tumor necrosis factor receptor superfamily member 10B	TNFRSF10B	0,736	nq
P05141	ADP/ATP translocase 2	SLC25A5	0,736	0,171
P30101	Protein disulfide-isomerase A3	PDIA3	0,731	0,208
P25705	ATP synthase subunit alpha, mitochondrial	ATP5A1	0,73	0,379
P38646	Stress-70 protein, mitochondrial	HSPA9	0,725	0,707

Q15149	plectin	PLEC	0,725	0,965
P49368	T-complex protein 1 subunit gamma	CCT3	0,72	0,242
P27797	Calreticulin	CALR	0,715	nq
Q5VZV1	Protein-lysine methyltransferase METTL21C	METTL21C	0,714	0,540
Q6EMK4	vasorin	VASN	0,712	0,474
P14625	Endoplasmin	HSP90B1	0,71	0,263
P48047	ATP synthase subunit O, mitochondrial	ATP5O	0,707	0,244
Q06830	peroxiredoxin-1	PRDX1	0,704	0,226
P06753	Tropomyosin alpha-3 chain	TPM3	0,697	0,110
Q99623	Prohibitin-2	PHB2	0,695	0,148
Q8NFZ8	Cell adhesion molecule 4	CADM4	0,694	0,428
O15061	Synemin	SYNM	0,692	0,244
P13667	Protein disulfide-isomerase A4	PDIA4	0,679	0,371
Q9Y6M7	Sodium bicarbonate cotransporter 3	SLC4A7	0,67	0,387
P68371	Tubulin beta-4B chain	TUBB4B	0,666	0,379
P62826	GTP-binding nuclear protein RAN	RAN	0,659	0,198
P07237	Protein disulfide-isomerase	P4HB	0,659	0,098
Q15365	Poly(RC)-binding protein 1	PCBP1	0,65	0,800
Q9Y2B0	Protein canopy homolog 2	CNPY2	0,649	nq
P12645	bone morphogenetic protein 3	BMP3	0,649	0,293
Q9BXB1	Leucine-rich repeat-containing G-protein coupled receptor 4	LGR4	0,648	0,059
Q14697	Neutral alpha-glucosidase AB	GANAB	0,647	0,414
P11498	pyruvate carboxylase, mitochondrial	PC	0,643	nq
P04899	Guanine nucleotide-binding protein G(i) subunit alpha-2	GNAI2	0,637	0,671
P23229	integrin alpha-6	ITGA6	0,636	0,243
P29317	Ephrin type-A receptor 2	EPHA2	0,629	0,460

P07204	Thrombomodulin	THBD	0,629	0,197
P23528	Cofilin-1	CFL1	0,625	0,412
P07099	epoxide hydrolase 1	EPHX1	0,62	0,116
P39656	Dolichyl-diphosphooligosaccharide--protein glycosyltransferase 48 kDa subunit	DDOST	0,62	0,075
P11021	78 kDa glucose-regulated protein	HSPA5	0,619	0,632
Q8TB96	T-cell immunomodulatory protein	ITFG1	0,615	0,139
P11142	Heat shock cognate 71 kDa protein	HSPA8	0,603	nq
P07355	Annexin A2	ANXA2	0,6	0,187
Q9Y624	Junctional adhesion molecule A	F11R	0,597	0,137
Q13444	Disintegrin and metalloproteinase domain-containing protein 15	ADAM15	0,595	0,129
P25205	DNA replication licensing factor mcm3	MCM3	0,588	0,226
P14314	Glucosidase 2 subunit beta	PRKCSH	0,587	nq
P10809	60 kDa heat shock protein, mitochondrial	HSPD1	0,582	0,393
P17342	Atrial natriuretic peptide receptor 3	NPR3	0,577	0,366
Q9NQC3	Reticulon-4	RTN4	0,573	0,095
P31943	Heterogeneous nuclear ribonucleoprotein H	HNRNPH1	0,56	0,669
P06703	protein S100-A6	S100A6	0,544	0,592
P05787	Keratin, type II cytoskeletal 8	KRT8	0,544	0,089
P04156	Major prion protein	PRNP	0,543	nq
P47756	F-actin-capping protein subunit beta	CAPZB	0,538	nq
P00558	phosphoglycerate kinase 1	PGK1	0,533	0,188
Q00610	Clathrin heavy chain 1	CLTC	0,531	0,235
P45880	Voltage-dependent anion-selective channel protein 2	VDAC2	0,53	0,117
P08238	Heat shock protein HSP 90-beta	HSP90AB1	0,528	0,231
P42704	Leucine-rich PPR motif-containing protein, mitochondrial	LRPPRC	0,523	0,421

P49411	elongation factor Tu, mitochondrial	TUFM	0,522	0,130
P60709	Actin, cytoplasmic 1	ACTB	0,515	0,139
P08581	Hepatocyte growth factor receptor	MET	0,513	0,299
Q13162	Peroxiredoxin-4	PRDX4	0,51	0,006
Q9BTM1	Histone H2A.J	H2AFJ	0,51	0,052
P46940	Ras GTPase-activating-like protein IQGAP1	IQGAP1	0,503	0,162
P60981	Destrin	DSTN	0,496	0,146
Q14444	Caprin-1	CAPRIN1	0,496	0,164
P08754	Guanine nucleotide-binding protein G(k) subunit alpha	GNAI3	0,494	0,135
P35232	Prohibitin	PHB	0,492	0,105
P30041	Peroxiredoxin-6	PRDX6	0,486	nq
Q86X29	Lipolysis-stimulated lipoprotein receptor	LSR	0,479	nq
Q92841	Probable ATP-dependent RNA helicase DDX17	DDX17	0,473	0,188
P04406	glyceraldehyde-3-phosphate dehydrogenase	GAPDH	0,466	0,044
P0DMV8	heat shock 70 kDa protein 1A	HSPA1A; HSPA1B	0,463	0,208
P63261	Actin, cytoplasmic 2	ACTG1	0,461	0,056
Q00325	Phosphate carrier protein, mitochondrial	SLC25A3	0,459	0,280
P68104	Elongation factor 1-alpha 1	EEF1A1	0,457	0,093
Q9Y277	Voltage-dependent anion-selective channel protein 3	VDAC3	0,45	0,342
P13639	Elongation factor 2	EEF2	0,447	0,007
P13797	Plastin-3	PLS3	0,429	0,070
O43707	Alpha-actinin-4	ACTN4	0,424	0,031
Q92598	Heat shock protein 105 kDa	HSPH1	0,423	0,151
P07900	Heat shock protein HSP 90-alpha	HSP90AA1	0,417	nq
P61978	Heterogeneous nuclear ribonucleoprotein K	HNRNPK	0,409	0,064
P30048	Thioredoxin-dependent peroxide reductase, mitochondrial	PRDX3	0,404	0,089

P16144	Integrin beta-4	ITGB4	0,402	0,158
O75369	Filamin-B	FLNB	0,391	nq
P14618	Pyruvate kinase PKM	PKM	0,389	0,135
Q8TEX9	Importin-4	IPO4	0,38	0,170
P06733	alpha-enolase	ENO1	0,365	nq
P55060	Exportin-2	CSE1L	0,362	0,072
P50990	T-complex protein 1 subunit theta	CCT8	0,328	nq
P52272	Heterogeneous nuclear ribonucleoprotein M	HNRNPM	0,326	0,179
P78527	DNA-dependent protein kinase catalytic subunit	PRKDC	0,311	0,150
Q16891	MICOS complex subunit Mic60	IMMT	0,284	0,215
P21333	Filamin-A	FLNA	0,28	0,218
P31946	14-3-3 protein beta/alpha	YWHAB	0,23	0,092
Q96QK1	Vacuolar protein sorting-associated protein 35	VPS35	0,221	nq
P56199	Integrin alpha-1	ITGA1	0,163	0,240
P52895	Aldo-keto reductase family 1 member C2	AKR1C2	0,07	0,140

Accession	Description	Gene symbol	Abundance Ratio: (WT, IFNγ) / (KO_IFITM1, IFNγ)	P-value
P13164	Interferon-induced transmembrane protein 1	IFITM1	12,032	0,015
P25942	Tumor necrosis factor receptor superfamily member 5	CD40	3,731	0,013
O14944	Proepiregulin	EREG	3,047	0,027
P20827	Ephrin-A1	EFNA1	2,961	0,006
Q9Y666	Solute carrier family 12 member 7	SLC12A7	2,757	nq
Q6PL18	ATPase family AAA domain-containing protein 2	ATAD2	2,61	0,076

Q92673	Sortilin-related receptor	SORL1	2,498	0,038
Q13740	CD166 antigen	ALCAM	2,304	0,014
P20645	Cation-dependent mannose-6-phosphate receptor	M6PR	2,269	0,011
P11717	Cation-independent mannose-6-phosphate receptor	IGF2R	2,242	0,003
P78310	Coxsackievirus and adenovirus receptor	CXADR	2,125	0,039
Q07954	prolow-density lipoprotein receptor-related protein 1	LRP1	2,07	0,113
P21589	5'-nucleotidase	NT5E	2,02	0,008
P78380	Oxidized low-density lipoprotein receptor 1	OLR1	1,987	0,073
Q5ZPR3	CD276 antigen	CD276	1,964	0,023
Q99523	Sortilin	SORT1	1,963	0,001
P01130	Low-density lipoprotein receptor	LDLR	1,922	0,005
P05534	HLA class I histocompatibility antigen, A-24 alpha chain	HLA-A	1,915	0,000
P30825	High affinity cationic amino acid transporter 1	SLC7A1	1,908	0,000
Q15043	Zinc transporter ZIP14	SLC39A14	1,833	0,964
Q29983	MHC class I polypeptide-related sequence A	MICA	1,804	0,158
P30479	HLA class I histocompatibility antigen, B-41 alpha chain	HLA-B	1,788	0,151
Q9H6X2	Anthrax toxin receptor 1	ANTXR1	1,784	0,363
P42892	Endothelin-converting enzyme 1	ECE1	1,751	0,372
P08648	Integrin alpha-5	ITGA5	1,749	0,022
P48509	CD151 antigen	CD151	1,74	0,358
Q10589	bone marrow stromal antigen 2	BST2	1,739	0,027
P18084	Integrin beta-5	ITGB5	1,723	0,165
O15230	Laminin subunit alpha-5	LAMA5	1,668	0,022
P13987	CD59 glycoprotein	CD59	1,651	0,665
P30486	HLA class I histocompatibility antigen, B-48 alpha chain	HLA-B	1,633	0,021
P04156	Major prion protein	PRNP	1,626	0,362

P43121	Cell surface glycoprotein MUC18	MCAM	1,61	0,080
P18827	syndecan-1	SDC1	1,6	0,305
P54709	sodium/potassium-transporting ATPase subunit beta-3	ATP1B3	1,586	0,026
P29323	Ephrin type-B receptor 2	EPHB2	1,584	0,061
P14209	CD99 antigen	CD99	1,561	0,137
O43278	Kunitz-type protease inhibitor 1	SPINT1	1,529	0,039
P60033	CD81 antigen	CD81	1,517	0,585
P23396	40S ribosomal protein S3	RPS3	1,512	0,028
P09382	Galectin-1	LGALS1	1,495	0,693
Q9HCJ1	progressive ankylosis protein homolog	ANKH	1,489	0,119
P08174	Complement decay-accelerating factor	CD55	1,487	0,054
P53794	sodium/myo-inositol cotransporter	SLC5A3	1,482	0,151
P54760	Ephrin type-B receptor 4	EPHB4	1,48	0,128
P55290	Cadherin-13	CDH13	1,465	0,132
Q13443	disintegrin and metalloproteinase domain-containing protein 9	ADAM9	1,465	0,219
Q99650	Oncostatin-M-specific receptor subunit beta	OSMR	1,454	0,149
P15941	Mucin-1	MUC1	1,447	0,121
Q9ULI3	Protein HEG homolog 1	HEG1	1,44	0,106
P09758	Tumor-associated calcium signal transducer 2	TACSTD2	1,436	0,222
Q9BZM5	UL16-binding protein 2	ULBP2	1,432	0,342
Q14126	Desmoglein-2	DSG2	1,429	0,519
Q8NFJ5	Retinoic acid-induced protein 3	GPRC5A	1,42	0,308
P06756	Integrin alpha-V	ITGAV	1,401	0,219
Q13308	Inactive tyrosine-protein kinase 7	PTK7	1,401	0,026
P15529	Membrane cofactor protein	CD46	1,39	0,238
P14384	Carboxypeptidase M	CPM	1,384	0,053

Q8IWA5	Choline transporter-like protein 2	SLC44A2	1,378	0,379
Q969P0	Immunoglobulin superfamily member 8	IGSF8	1,372	0,673
Q04941	proteolipid protein 2	PLP2	1,371	0,339
Q9P2B2	prostaglandin F2 receptor negative regulator	PTGFRN	1,366	0,143
P14923	Junction plakoglobin	JUP	1,362	0,756
Q96J84	Kin of IRRE-like protein 1	KIRREL; KIRREL1	1,343	0,128
Q86V24	Adiponectin receptor protein 2	ADIPOR2	1,339	0,299
O14672	Disintegrin and metalloproteinase domain-containing protein 10	ADAM10	1,338	0,228
Q9NPH3	Interleukin-1 receptor accessory protein	IL1RAP	1,333	0,119
Q12913	Receptor-type tyrosine-protein phosphatase eta	PTPRJ	1,324	0,154
P16070	CD44 antigen	CD44	1,317	0,476
P25205	DNA replication licensing factor mcm3	MCM3	1,316	nq
P30530	Tyrosine-protein kinase receptor UFO	AXL	1,315	0,119
P41440	Folate transporter 1	SLC19A1	1,303	0,254
Q96NT5	Proton-coupled folate transporter	SLC46A1	1,3	0,979
P53985	Monocarboxylate transporter 1	SLC16A1	1,3	0,004
P08754	Guanine nucleotide-binding protein G(k) subunit alpha	GNAI3	1,295	nq
Q8IWT6	volume-regulated anion channel subunit LRRC8A	LRRC8A	1,293	0,499
P48960	CD97 antigen	CD97; ADGRE5	1,284	0,365
P49184	Deoxyribonuclease-1-like 1	DNASE1L1	1,282	0,465
Q9NUM4	Transmembrane protein 106B	TMEM106B	1,276	0,392
Q92542	Nicastrin	NCSTN	1,274	0,618
P26006	Integrin alpha-3	ITGA3	1,271	0,067
Q13332	Receptor-type tyrosine-protein phosphatase S	PTPRS	1,269	0,247
P17813	Endoglin	ENG	1,263	0,015
Q9UIQ6	Leucyl-cystinyl aminopeptidase	LNPEP	1,26	0,541

Q9H2H9	Sodium-coupled neutral amino acid transporter 1	SLC38A1	1,253	0,255
P02786	Transferrin receptor protein 1	TFRC	1,246	0,664
Q12907	Vesicular integral-membrane protein VIP36	LMAN2	1,245	0,464
P31641	Sodium- and chloride-dependent taurine transporter	SLC6A6	1,24	0,574
O15031	Plexin-B2	PLXNB2	1,235	0,132
Q92692	Nectin-2	PVRL2; NECTIN2	1,233	0,546
P05023	Sodium/potassium-transporting ATPase subunit alpha-1	ATP1A1	1,23	0,007
P04626	Receptor tyrosine-protein kinase erbB-2	ERBB2	1,227	0,056
Q6NSJ0	Uncharacterized family 31 glucosidase KIAA1161	KIAA1161; MYORG	1,225	0,040
Q13641	Trophoblast glycoprotein	TPBG	1,223	0,166
Q9NV96	Cell cycle control protein 50A	TMEM30A	1,223	0,335
Q9UNN8	Endothelial protein C receptor	PROCR	1,216	0,433
P33527	Multidrug resistance-associated protein 1	ABCC1	1,206	0,511
Q08722	Leukocyte surface antigen CD47	CD47	1,202	0,104
Q6P4Q7	metal transporter CNNM4	CNNM4	1,202	0,239
P15529	Membrane cofactor protein	CD46	1,201	0,442
P08729	Keratin, type II cytoskeletal 7	KRT7	1,19	0,387
P35613	Basigin	BSG	1,179	0,126
Q13444	Disintegrin and metalloproteinase domain-containing protein 15	ADAM15	1,164	0,118
P27824	Calnexin	CANX	1,163	0,211
P05026	Sodium/potassium-transporting ATPase subunit beta-1	ATP1B1	1,161	0,440
P43007	neutral amino acid transporter A	SLC1A4	1,156	0,009
B7Z368	Uncharacterized protein C10orf142	LOC100130539; C10orf142	1,148	0,533
P0C854	Putative cat eye syndrome critical region protein 9	CECR9	1,146	0,420
Q92859	neogenin	NEO1	1,144	0,642
P11279	Lysosome-associated membrane glycoprotein 1	LAMP1	1,144	0,465

P62826	GTP-binding nuclear protein RAN	RAN	1,131	0,932
O14763	Tumor necrosis factor receptor superfamily member 10B	TNFRSF10B	1,13	0,359
P15151	Poliovirus receptor	PVR	1,125	0,573
Q9UIW2	Plexin-A1	PLXNA1	1,119	0,686
Q16563	Synaptophysin-like protein 1	SYPL1	1,115	0,893
P04843	Dolichyl-diphosphooligosaccharide--protein glycosyltransferase subunit 1	RPN1	1,114	0,357
Q6YHK3	CD109 antigen	CD109	1,1	0,362
Q5RHP9	glutamate-rich protein 3	C1orf173; ERICH3	1,1	0,153
P05556	Integrin beta-1	ITGB1	1,097	0,524
P46782	40S ribosomal protein S5	RPS5	1,091	0,512
P08962	CD63 antigen	CD63	1,086	nq
Q15365	Poly(RC)-binding protein 1	PCBP1	1,077	0,910
P11166	Solute carrier family 2, facilitated glucose transporter member 1	SLC2A1	1,07	0,607
P61978	Heterogeneous nuclear ribonucleoprotein K	HNRNPK	1,067	0,652
O75144	ICOS ligand	ICOSLG; LOC102723996	1,063	nq
P35052	Glypican-1	GPC1	1,061	0,373
P63000	Ras-related C3 botulinum toxin substrate 1	RAC1	1,061	0,739
P50281	Matrix metalloproteinase-14	MMP14	1,059	0,794
P15328	Folate receptor alpha	FOLR1	1,058	0,876
P19256	Lymphocyte function-associated antigen 3	CD58	1,053	0,885
O15431	High affinity copper uptake protein 1	SLC31A1	1,046	0,612
Q04695	Keratin, type I cytoskeletal 17	KRT17	1,044	0,530
Q9NQC3	Reticulon-4	RTN4	1,041	0,917
Q14118	Dystroglycan	DAG1	1,033	0,842
Q9Y2B0	Protein canopy homolog 2	CNPY2	1,032	0,138
Q06830	peroxiredoxin-1	PRDX1	1,03	nq

P25445	Tumor necrosis factor receptor superfamily member 6	FAS	1,021	0,786
P30048	Thioredoxin-dependent peroxide reductase, mitochondrial	PRDX3	1,012	nq
P98172	ephrin-B1	EFNB1	1,01	nq
Q04721	Neurogenic locus notch homolog protein 2	NOTCH2	1,008	0,599
Q92973	transportin-1	TNPO1	1,007	0,987
P61769	Beta-2-microglobulin	B2M	0,997	0,723
P16615	Sarcoplasmic/endoplasmic reticulum calcium ATPase 2	ATP2A2	0,996	0,447
P43307	Translocon-associated protein subunit alpha	SSR1	0,992	0,328
P12645	bone morphogenetic protein 3	BMP3	0,99	nq
P50454	Serpin H1	SERPINH1	0,985	0,829
P10586	Receptor-type tyrosine-protein phosphatase F	PTPRF	0,984	0,619
A5PL33	protein KRBA1	KRBA1	0,981	0,252
P48047	ATP synthase subunit O, mitochondrial	ATP5O	0,981	0,882
P08069	Insulin-like growth factor 1 receptor	IGF1R	0,981	0,053
P78324	Tyrosine-protein phosphatase non-receptor type substrate 1	SIRPA	0,979	0,935
P09622	Dihydrolipoyl dehydrogenase, mitochondrial	DLD	0,974	0,853
Q687X5	Metalloreductase STEAP4	STEAP4	0,972	nq
P31431	syndecan-4	SDC4	0,97	0,676
Q9Y289	Sodium-dependent multivitamin transporter	SLC5A6	0,964	0,991
Q9Y6M5	zinc transporter 1	SLC30A1	0,963	0,530
P47756	F-actin-capping protein subunit beta	CAPZB	0,957	0,507
P51149	ras-related protein Rab-7a	RAB7A	0,955	0,234
P11498	pyruvate carboxylase, mitochondrial	PC	0,952	0,427
O00592	Podocalyxin	PODXL	0,952	0,861
P13726	tissue factor	F3	0,947	0,849
Q8WWI5	choline transporter-like protein 1	SLC44A1	0,943	0,812

P23634	Plasma membrane calcium-transporting ATPase 4	ATP2B4	0,937	0,984
Q04912	Macrophage-stimulating protein receptor	MST1R	0,935	0,444
P21926	CD9 antigen	CD9	0,935	0,606
O15427	Monocarboxylate transporter 4	SLC16A3	0,932	0,901
P05362	Intercellular adhesion molecule 1	ICAM1	0,931	0,567
P31943	Heterogeneous nuclear ribonucleoprotein H	HNRNPH1	0,93	0,797
Q7L1W4	volume-regulated anion channel subunit LRRC8D	LRRC8D	0,904	0,681
P50895	Basal cell adhesion molecule	BCAM	0,901	0,323
P32004	Neural cell adhesion molecule L1	L1CAM	0,896	0,916
P04222	HLA class I histocompatibility antigen, Cw-3 alpha chain	HLA-C	0,894	0,281
Q96QD8	sodium-coupled neutral amino acid transporter 2	SLC38A2	0,893	0,011
P17301	Integrin alpha-2	ITGA2	0,891	0,348
P00533	epidermal growth factor receptor	EGFR	0,887	0,221
P68363	Tubulin alpha-1B chain	TUBA1B	0,883	0,147
P07237	Protein disulfide-isomerase	P4HB	0,88	0,371
Q15084	Protein disulfide-isomerase A6	PDIA6	0,877	0,729
Q13421	Mesothelin	MSLN	0,871	0,282
P06576	ATP synthase subunit beta, mitochondrial	ATP5B	0,868	0,905
P04406	glyceraldehyde-3-phosphate dehydrogenase	GAPDH	0,862	0,044
Q8NE01	Metal transporter CNNM3	CNNM3	0,857	0,754
O95297	Myelin protein zero-like protein 1	MPZL1	0,853	0,665
Q15262	Receptor-type tyrosine-protein phosphatase kappa	PTPRK	0,849	0,058
Q15388	Mitochondrial import receptor subunit TOM20 homolog	TOMM20	0,848	0,410
P06733	alpha-enolase	ENO1	0,847	0,744
Q92598	Heat shock protein 105 kDa	HSPH1	0,841	0,823
Q9Y639	Neuroplastin	NPTN	0,838	0,800

P38646	Stress-70 protein, mitochondrial	HSPA9	0,823	0,255
Q9Y624	Junctional adhesion molecule A	F11R	0,816	0,905
P08195	4F2 cell-surface antigen heavy chain	SLC3A2	0,8	0,426
P78527	DNA-dependent protein kinase catalytic subunit	PRKDC	0,799	0,052
P08571	Monocyte differentiation antigen CD14	CD14	0,798	0,281
P05187	alkaline phosphatase, placental type	ALPP	0,796	0,437
Q03405	Urokinase plasminogen activator surface receptor	PLAUR	0,795	0,133
P09603	Macrophage colony-stimulating factor 1	CSF1	0,792	0,037
P07355	Annexin A2	ANXA2	0,781	0,691
P68371	Tubulin beta-4B chain	TUBB4B	0,778	0,005
P14625	Endoplasmin	HSP90B1	0,776	0,391
P27797	Calreticulin	CALR	0,774	0,144
P17342	Atrial natriuretic peptide receptor 3	NPR3	0,773	0,662
Q15758	Neutral amino acid transporter B(0)	SLC1A5	0,773	0,227
Q00325	Phosphate carrier protein, mitochondrial	SLC25A3	0,77	0,768
P04899	Guanine nucleotide-binding protein G(i) subunit alpha-2	GNAI2	0,768	0,595
P60981	Destrin	DSTN	0,768	0,379
P21333	Filamin-A	FLNA	0,761	0,001
P23229	integrin alpha-6	ITGA6	0,76	0,387
Q15149	plectin	PLEC	0,759	0,511
P13667	Protein disulfide-isomerase A4	PDIA4	0,757	0,085
Q8WTV0	Scavenger receptor class B member 1	SCARB1	0,756	0,451
P35232	Prohibitin	PHB	0,753	0,259
P62979	Ubiquitin-40S ribosomal protein S27a	RPS27A	0,744	0,082
P11142	Heat shock cognate 71 kDa protein	HSPA8	0,743	0,012
P06753	Tropomyosin alpha-3 chain	TPM3	0,742	0,093

P25705	ATP synthase subunit alpha, mitochondrial	ATP5A1	0,741	0,079
P46940	Ras GTPase-activating-like protein IQGAP1	IQGAP1	0,733	0,021
O00571	ATP-dependent RNA helicase DDX3X	DDX3X	0,733	0,106
Q8WVN6	Secreted and transmembrane protein 1	SECTM1	0,73	0,020
P05141	ADP/ATP translocase 2	SLC25A5	0,729	0,146
P45880	Voltage-dependent anion-selective channel protein 2	VDAC2	0,727	0,383
Q9NZJ5	Eukaryotic translation initiation factor 2-alpha kinase 3	EIF2AK3	0,717	0,017
Q9UJS0	Calcium-binding mitochondrial carrier protein Aralar2	SLC25A13	0,717	nq
P07437	tubulin beta chain	TUBB	0,716	nq
P68104	Elongation factor 1-alpha 1	EEF1A1	0,715	0,398
P04844	Dolichyl-diphosphooligosaccharide--protein glycosyltransferase subunit 2	RPN2	0,714	0,006
P14618	Pyruvate kinase PKM	PKM	0,709	0,479
Q15417	Calponin-3	CNN3	0,702	0,070
Q4KMQ2	Anoctamin-6	ANO6	0,702	0,089
Q02978	Mitochondrial 2-oxoglutarate/malate carrier protein	SLC25A11	0,702	0,243
P18433	Receptor-type tyrosine-protein phosphatase alpha	PTPRA	0,702	nq
Q3ZCN5	Otogelin-like protein	OTOGL	0,701	nq
P00558	phosphoglycerate kinase 1	PGK1	0,698	nq
Q01650	large neutral amino acids transporter small subunit 1	SLC7A5	0,697	0,335
P05787	Keratin, type II cytoskeletal 8	KRT8	0,691	0,026
P31327	Carbamoyl-phosphate synthase [ammonia], mitochondrial	CPS1	0,688	0,709
Q9Y277	Voltage-dependent anion-selective channel protein 3	VDAC3	0,685	0,007
P60709	Actin, cytoplasmic 1	ACTB	0,68	0,091
P0DMV8	heat shock 70 kDa protein 1A	HSPA1A; HSPA1B	0,674	0,205
Q99714	3-hydroxyacyl-CoA dehydrogenase type-2	HSD17B10	0,671	0,125
Q03167	Transforming growth factor beta receptor type 3	TGFBR3	0,671	nq

P22695	Cytochrome b-c1 complex subunit 2, mitochondrial	UQCRC2	0,67	0,343
P07099	epoxide hydrolase 1	EPHX1	0,663	0,585
Q9H5V8	CUB domain-containing protein 1	CDCP1	0,662	0,133
P29317	Ephrin type-A receptor 2	EPHA2	0,659	0,091
Q8NFZ8	Cell adhesion molecule 4	CADM4	0,658	0,439
P13639	Elongation factor 2	EEF2	0,657	0,205
Q9P035	Very-long-chain (3R)-3-hydroxyacyl-CoA dehydratase 3	PTPLAD1; HACD3	0,656	0,033
Q14697	Neutral alpha-glucosidase AB	GANAB	0,651	nq
P11586	C-1-tetrahydrofolate synthase, cytoplasmic	MTHFD1	0,649	nq
P50990	T-complex protein 1 subunit theta	CCT8	0,649	nq
P55060	Exportin-2	CSE1L	0,649	0,450
P42224	Signal transducer and activator of transcription 1-alpha/beta	STAT1	0,647	0,010
Q14444	Caprin-1	CAPRIN1	0,643	0,045
P04920	Anion exchange protein 2	SLC4A2	0,635	nq
P30101	Protein disulfide-isomerase A3	PDIA3	0,634	nq
P06703	protein S100-A6	S100A6	0,632	0,428
P23528	Cofilin-1	CFL1	0,632	0,241
P08195	4F2 cell-surface antigen heavy chain	SLC3A2	0,631	0,135
P42704	Leucine-rich PPR motif-containing protein, mitochondrial	LRPPRC	0,63	0,036
P32119	Peroxiredoxin-2	PRDX2	0,626	0,515
Q16891	MICOS complex subunit Mic60	IMMT	0,623	0,278
P07204	Thrombomodulin	THBD	0,622	0,360
Q99623	Prohibitin-2	PHB2	0,618	nq
P08238	Heat shock protein HSP 90-beta	HSP90AB1	0,611	nq
Q13162	Peroxiredoxin-4	PRDX4	0,61	0,288
P21796	voltage-dependent anion-selective channel protein 1	VDAC1	0,61	0,252

Q86X29	Lipolysis-stimulated lipoprotein receptor	LSR	0,609	0,209
P07737	profilin-1	PFN1	0,605	0,035
P63261	Actin, cytoplasmic 2	ACTG1	0,598	0,245
P11021	78 kDa glucose-regulated protein	HSPA5	0,591	0,347
P14314	Glucosidase 2 subunit beta	PRKCSH	0,59	0,360
Q9Y6M7	Sodium bicarbonate cotransporter 3	SLC4A7	0,59	0,177
P30041	Peroxiredoxin-6	PRDX6	0,589	0,148
Q6EMK4	vasorin	VASN	0,579	0,080
Q92841	Probable ATP-dependent RNA helicase DDX17	DDX17	0,572	0,064
P08581	Hepatocyte growth factor receptor	MET	0,569	0,054
P10809	60 kDa heat shock protein, mitochondrial	HSPD1	0,564	0,151
P16144	Integrin beta-4	ITGB4	0,562	0,000
P23284	peptidyl-prolyl cis-trans isomerase B	PPIB	0,551	0,800
P23381	Tryptophan--tRNA ligase, cytoplasmic	WARS	0,548	0,043
P39656	Dolichyl-diphosphooligosaccharide--protein glycosyltransferase 48 kDa subunit	DDOST	0,544	nq
O43707	Alpha-actinin-4	ACTN4	0,519	0,053
P52272	Heterogeneous nuclear ribonucleoprotein M	HNRNPM	0,517	0,180
P07900	Heat shock protein HSP 90-alpha	HSP90AA1	0,514	0,117
O75369	Filamin-B	FLNB	0,489	0,043
P56199	Integrin alpha-1	ITGA1	0,474	0,254
Q9BTM1	Histone H2A.J	H2AFJ	0,415	0,133
Q8TEX9	Importin-4	IPO4	0,407	0,086
O15061	Synemin	SYNM	0,362	nq
P30153	serine/threonine-protein phosphatase 2A 65 kDa regulatory subunit A alpha isoform	PPP2R1A	0,353	0,187

Q5VZV1	Protein-lysine methyltransferase METTL21C	METTL21C	0,338	nq
P52895	Aldo-keto reductase family 1 member C2	AKR1C2	0,336	0,006
P08758	annexin A5	ANXA5	0,333	nq
Q00610	Clathrin heavy chain 1	CLTC	0,333	0,033
Q96QK1	Vacuolar protein sorting-associated protein 35	VPS35	0,275	0,304
Q9BXB1	Leucine-rich repeat-containing G-protein coupled receptor 4	LGR4	0,274	nq
P13797	Plastin-3	PLS3	0,272	nq
P62937	peptidyl-prolyl cis-trans isomerase A	PPIA	0,202	nq
Q8TCZ2	CD99 antigen-like protein 2	CD99L2	0,108	0,086

Accession	Description	Gene symbol	Abundance Ratio: (WT, IFNγ) / (WT, ctrl)	P-value
P42224	Signal transducer and activator of transcription 1-alpha/beta	STAT1	3,313	0,008
P30479	HLA class I histocompatibility antigen, B-41 alpha chain	HLA-B	2,602	0,001
P13164	Interferon-induced transmembrane protein 1	IFITM1	2,29	0,017
P05362	Intercellular adhesion molecule 1	ICAM1	2,21	0,013
P23381	Tryptophan--tRNA ligase, cytoplasmic	WARS	2,098	0,301
P30486	HLA class I histocompatibility antigen, B-48 alpha chain	HLA-B	2,09	0,010
P04222	HLA class I histocompatibility antigen, Cw-3 alpha chain	HLA-C	2,041	0,000
P56199	Integrin alpha-1	ITGA1	1,969	nq
P61769	Beta-2-microglobulin	B2M	1,926	0,085
P08754	Guanine nucleotide-binding protein G(k) subunit alpha	GNAI3	1,752	nq
P05534	HLA class I histocompatibility antigen, A-24 alpha chain	HLA-A	1,682	0,000
P21589	5'-nucleotidase	NT5E	1,671	0,058
P25942	Tumor necrosis factor receptor superfamily member 5	CD40	1,605	0,031

Q8WVN6	Secreted and transmembrane protein 1	SECTM1	1,529	0,005
P09622	Dihydrolipoyl dehydrogenase, mitochondrial	DLD	1,518	0,763
P78380	Oxidized low-density lipoprotein receptor 1	OLR1	1,512	nq
Q08722	Leukocyte surface antigen CD47	CD47	1,497	0,143
P09603	Macrophage colony-stimulating factor 1	CSF1	1,478	0,011
P04156	Major prion protein	PRNP	1,464	0,050
P15941	Mucin-1	MUC1	1,454	0,433
Q14697	Neutral alpha-glucosidase AB	GANAB	1,438	0,062
Q00325	Phosphate carrier protein, mitochondrial	SLC25A3	1,428	nq
P30048	Thioredoxin-dependent peroxide reductase, mitochondrial	PRDX3	1,421	0,054
O14672	Disintegrin and metalloproteinase domain-containing protein 10	ADAM10	1,407	nq
P13987	CD59 glycoprotein	CD59	1,373	0,283
P48047	ATP synthase subunit O, mitochondrial	ATP5O	1,365	0,374
Q10589	bone marrow stromal antigen 2	BST2	1,363	0,360
Q86V24	Adiponectin receptor protein 2	ADIPOR2	1,344	0,276
P11279	Lysosome-associated membrane glycoprotein 1	LAMP1	1,341	0,876
P47756	F-actin-capping protein subunit beta	CAPZB	1,308	0,262
Q5ZPR3	CD276 antigen	CD276	1,3	0,875
P62937	peptidyl-prolyl cis-trans isomerase A	PPIA	1,299	0,063
P60033	CD81 antigen	CD81	1,294	nq
P62826	GTP-binding nuclear protein RAN	RAN	1,283	0,950
Q9ULI3	Protein HEG homolog 1	HEG1	1,268	0,263
Q15365	Poly(RC)-binding protein 1	PCBP1	1,267	0,318
P16144	Integrin beta-4	ITGB4	1,256	nq
P16070	CD44 antigen	CD44	1,25	nq
P14923	Junction plakoglobin	JUP	1,247	0,157

P14209	CD99 antigen	CD99	1,246	0,331
P08962	CD63 antigen	CD63	1,241	0,290
Q15043	Zinc transporter ZIP14	SLC39A14	1,232	0,633
Q13444	Disintegrin and metalloproteinase domain-containing protein 15	ADAM15	1,231	0,646
O14944	Proepiregulin	EREG	1,228	0,363
Q86X29	Lipolysis-stimulated lipoprotein receptor	LSR	1,227	0,441
P61978	Heterogeneous nuclear ribonucleoprotein K	HNRNPK	1,221	0,170
P23396	40S ribosomal protein S3	RPS3	1,207	nq
Q9H6X2	Anthrax toxin receptor 1	ANTXR1	1,201	0,166
P27797	Calreticulin	CALR	1,2	0,136
Q15262	Receptor-type tyrosine-protein phosphatase kappa	PTPRK	1,194	0,083
P07099	epoxide hydrolase 1	EPHX1	1,193	0,022
P46940	Ras GTPase-activating-like protein IQGAP1	IQGAP1	1,176	0,952
Q9Y639	Neuroplastin	NPTN	1,176	0,817
Q06830	peroxiredoxin-1	PRDX1	1,175	0,671
P04899	Guanine nucleotide-binding protein G(i) subunit alpha-2	GNAI2	1,169	0,196
P31431	syndecan-4	SDC4	1,165	0,610
P63000	Ras-related C3 botulinum toxin substrate 1	RAC1	1,163	0,932
P17813	Endoglin	ENG	1,162	0,188
O14763	Tumor necrosis factor receptor superfamily member 10B	TNFRSF10B	1,161	0,384
Q9H5V8	CUB domain-containing protein 1	CDCP1	1,16	0,536
Q9UNN8	Endothelial protein C receptor	PROCR	1,157	0,361
Q14126	Desmoglein-2	DSG2	1,155	0,853
O00592	Podocalyxin	PODXL	1,155	0,092
Q99714	3-hydroxyacyl-CoA dehydrogenase type-2	HSD17B10	1,148	0,424
P42704	Leucine-rich PPR motif-containing protein, mitochondrial	LRPPRC	1,147	nq

P50990	T-complex protein 1 subunit theta	CCT8	1,142	0,332
P09382	Galectin-1	LGALS1	1,136	0,502
P04844	Dolichyl-diphosphooligosaccharide--protein glycosyltransferase subunit 2	RPN2	1,126	0,392
P63261	Actin, cytoplasmic 2	ACTG1	1,125	nq
P08581	Hepatocyte growth factor receptor	MET	1,125	0,537
Q16891	MICOS complex subunit Mic60	IMMT	1,121	0,218
P29323	Ephrin type-B receptor 2	EPHB2	1,12	nq
P04626	Receptor tyrosine-protein kinase erbB-2	ERBB2	1,12	0,194
Q8IWT6	volume-regulated anion channel subunit LRRC8A	LRRC8A	1,119	0,149
O15230	Laminin subunit alpha-5	LAMA5	1,1	0,408
Q6NSJ0	Uncharacterized family 31 glucosidase KIAA1161	KIAA1161; MYORG	1,096	0,519
P13667	Protein disulfide-isomerase A4	PDIA4	1,096	0,768
P50454	Serpin H1	SERPINH1	1,095	0,717
Q15149	plectin	PLEC	1,094	0,567
Q6PL18	ATPase family AAA domain-containing protein 2	ATAD2	1,093	0,127
P48509	CD151 antigen	CD151	1,088	0,753
P05141	ADP/ATP translocase 2	SLC25A5	1,088	0,880
P68104	Elongation factor 1-alpha 1	EEF1A1	1,087	0,625
Q92859	neogenin	NEO1	1,086	0,617
Q13332	Receptor-type tyrosine-protein phosphatase S	PTPRS	1,085	0,493
P05556	Integrin beta-1	ITGB1	1,085	0,679
P15529	Membrane cofactor protein	CD46	1,084	0,199
P32004	Neural cell adhesion molecule L1	L1CAM	1,084	0,578
P62979	Ubiquitin-40S ribosomal protein S27a	RPS27A	1,083	0,472
Q92692	Nectin-2	PVRL2; NECTIN2	1,077	0,339
P35232	Prohibitin	PHB	1,073	0,287

Q9Y2B0	Protein canopy homolog 2	CNPY2	1,069	0,662
Q8IWA5	Choline transporter-like protein 2	SLC44A2	1,062	nq
Q92598	Heat shock protein 105 kDa	HSPH1	1,062	0,931
P17342	Atrial natriuretic peptide receptor 3	NPR3	1,06	0,792
Q9Y6M7	Sodium bicarbonate cotransporter 3	SLC4A7	1,06	0,522
Q04912	Macrophage-stimulating protein receptor	MST1R	1,056	0,825
Q14118	Dystroglycan	DAG1	1,053	0,886
O75144	ICOS ligand	ICOSLG; LOC102723996	1,053	0,483
P31641	Sodium- and chloride-dependent taurine transporter	SLC6A6	1,051	0,248
P78527	DNA-dependent protein kinase catalytic subunit	PRKDC	1,05	0,391
P26006	Integrin alpha-3	ITGA3	1,05	0,836
Q04941	proteolipid protein 2	PLP2	1,045	0,224
P11717	Cation-independent mannose-6-phosphate receptor	IGF2R	1,031	0,764
Q9UIW2	Plexin-A1	PLXNA1	1,03	0,614
Q9HCJ1	progressive ankylosis protein homolog	ANKH	1,028	0,980
P18084	Integrin beta-5	ITGB5	1,023	0,778
P35613	Basigin	BSG	1,023	0,844
Q13162	Peroxiredoxin-4	PRDX4	1,021	0,725
O43278	Kunitz-type protease inhibitor 1	SPINT1	1,013	0,921
Q03405	Urokinase plasminogen activator surface receptor	PLAUR	1,01	0,460
P18827	syndecan-1	SDC1	1,009	0,210
P45880	Voltage-dependent anion-selective channel protein 2	VDAC2	1,005	0,421
P15151	Poliovirus receptor	PVR	1,004	0,835
P30041	Peroxiredoxin-6	PRDX6	1,004	0,979
Q5RHP9	glutamate-rich protein 3	C1orf173; ERICH3	1,004	0,958
P12645	bone morphogenetic protein 3	BMP3	1,003	0,903

Q9Y6M5	zinc transporter 1	SLC30A1	1,001	0,991
P08174	Complement decay-accelerating factor	CD55	0,992	0,448
P05023	Sodium/potassium-transporting ATPase subunit alpha-1	ATP1A1	0,992	0,971
Q04721	Neurogenic locus notch homolog protein 2	NOTCH2	0,991	0,979
P29317	Ephrin type-A receptor 2	EPHA2	0,991	0,036
O95297	Myelin protein zero-like protein 1	MPZL1	0,986	0,160
P27824	Calnexin	CANX	0,986	0,940
P15529	Membrane cofactor protein	CD46	0,984	0,625
P48960	CD97 antigen	CD97; ADGRE5	0,981	0,865
P23229	integrin alpha-6	ITGA6	0,979	0,505
P42892	Endothelin-converting enzyme 1	ECE1	0,978	0,952
Q96J84	Kin of IRRE-like protein 1	KIRREL; KIRREL1	0,976	0,438
P43307	Translocon-associated protein subunit alpha	SSR1	0,973	0,138
Q99650	Oncostatin-M-specific receptor subunit beta	OSMR	0,969	nq
O15031	Plexin-B2	PLXNB2	0,969	0,965
Q92542	Nicastrin	NCSTN	0,969	0,477
P31946	14-3-3 protein beta/alpha	YWHAB	0,968	0,482
A5PL33	protein KRBA1	KRBA1	0,966	nq
P53985	Monocarboxylate transporter 1	SLC16A1	0,961	0,611
P04843	Dolichyl-diphosphooligosaccharide--protein glycosyltransferase subunit 1	RPN1	0,959	0,186
P10586	Receptor-type tyrosine-protein phosphatase F	PTPRF	0,958	0,836
Q04695	Keratin, type I cytoskeletal 17	KRT17	0,957	0,952
P07204	Thrombomodulin	THBD	0,957	0,976
Q9P2B2	prostaglandin F2 receptor negative regulator	PTGFRN	0,956	nq
P52272	Heterogeneous nuclear ribonucleoprotein M	HNRNPM	0,956	0,431
P30530	Tyrosine-protein kinase receptor UFO	AXL	0,954	0,641

Q9Y289	Sodium-dependent multivitamin transporter	SLC5A6	0,952	0,807
P14384	Carboxypeptidase M	CPM	0,951	0,652
Q9H2H9	Sodium-coupled neutral amino acid transporter 1	SLC38A1	0,949	0,410
Q13308	Inactive tyrosine-protein kinase 7	PTK7	0,946	0,848
Q7L1W4	volume-regulated anion channel subunit LRRC8D	LRRC8D	0,946	0,468
P01130	Low-density lipoprotein receptor	LDLR	0,945	0,500
P78310	Coxsackievirus and adenovirus receptor	CXADR	0,944	0,450
Q9NQC3	Reticulon-4	RTN4	0,934	0,188
P33527	Multidrug resistance-associated protein 1	ABCC1	0,932	0,922
P05026	Sodium/potassium-transporting ATPase subunit beta-1	ATP1B1	0,932	0,498
Q9NV96	Cell cycle control protein 50A	TMEM30A	0,926	0,825
P25705	ATP synthase subunit alpha, mitochondrial	ATP5A1	0,924	0,943
P98172	ephrin-B1	EFNB1	0,923	0,087
P07355	Annexin A2	ANXA2	0,922	0,973
Q969P0	Immunoglobulin superfamily member 8	IGSF8	0,922	0,219
P54709	sodium/potassium-transporting ATPase subunit beta-3	ATP1B3	0,922	0,505
P14618	Pyruvate kinase PKM	PKM	0,921	0,770
P49184	Deoxyribonuclease-1-like 1	DNASE1L1	0,92	0,572
P00533	epidermal growth factor receptor	EGFR	0,916	0,688
O15427	Monocarboxylate transporter 4	SLC16A3	0,916	0,696
P08648	Integrin alpha-5	ITGA5	0,915	0,075
Q96NT5	Proton-coupled folate transporter	SLC46A1	0,911	0,285
Q9Y624	Junctional adhesion molecule A	F11R	0,91	0,276
Q8NFZ8	Cell adhesion molecule 4	CADM4	0,905	0,724
P14314	Glucosidase 2 subunit beta	PRKCSH	0,905	0,439
Q12913	Receptor-type tyrosine-protein phosphatase eta	PTPRJ	0,904	0,604

Q12907	Vesicular integral-membrane protein VIP36	LMAN2	0,904	0,498
P11021	78 kDa glucose-regulated protein	HSPA5	0,898	0,902
P10809	60 kDa heat shock protein, mitochondrial	HSPD1	0,894	0,200
P50895	Basal cell adhesion molecule	BCAM	0,893	0,267
Q687X5	Metalloreductase STEAP4	STEAP4	0,89	0,930
P20827	Ephrin-A1	EFNA1	0,89	0,489
P0DMV8	heat shock 70 kDa protein 1A	HSPA1A; HSPA1B	0,889	0,590
P38646	Stress-70 protein, mitochondrial	HSPA9	0,887	0,042
P68363	Tubulin alpha-1B chain	TUBA1B	0,882	0,531
Q6YHK3	CD109 antigen	CD109	0,878	0,349
P39656	Dolichyl-diphosphooligosaccharide--protein glycosyltransferase 48 kDa subunit	DDOST	0,871	0,195
P05787	Keratin, type II cytoskeletal 8	KRT8	0,87	0,224
P14625	Endoplasmin	HSP90B1	0,866	nq
Q9BZM5	UL16-binding protein 2	ULBP2	0,865	0,930
P04406	glyceraldehyde-3-phosphate dehydrogenase	GAPDH	0,863	0,118
Q8WWI5	choline transporter-like protein 1	SLC44A1	0,863	0,026
P22695	Cytochrome b-c1 complex subunit 2, mitochondrial	UQCRC2	0,863	nq
P43121	Cell surface glycoprotein MUC18	MCAM	0,863	0,782
P60709	Actin, cytoplasmic 1	ACTB	0,862	0,700
P06703	protein S100-A6	S100A6	0,856	0,473
P20645	Cation-dependent mannose-6-phosphate receptor	M6PR	0,855	0,857
O75369	Filamin-B	FLNB	0,854	0,159
Q92841	Probable ATP-dependent RNA helicase DDX17	DDX17	0,849	nq
P11142	Heat shock cognate 71 kDa protein	HSPA8	0,849	0,318
Q9NPH3	Interleukin-1 receptor accessory protein	IL1RAP	0,848	0,804

Q8WTV0	Scavenger receptor class B member 1	SCARB1	0,844	0,721
P60981	Destrin	DSTN	0,843	0,466
Q13421	Mesothelin	MSLN	0,843	0,179
Q16563	Synaptophysin-like protein 1	SYPL1	0,84	0,133
P02786	Transferrin receptor protein 1	TFRC	0,839	0,515
P50281	Matrix metalloproteinase-14	MMP14	0,839	0,108
Q92673	Sortilin-related receptor	SORL1	0,839	0,199
P06756	Integrin alpha-V	ITGAV	0,835	0,145
P30101	Protein disulfide-isomerase A3	PDIA3	0,835	0,007
Q6EMK4	vasorin	VASN	0,834	0,882
P05187	alkaline phosphatase, placental type	ALPP	0,831	0,486
Q01650	large neutral amino acids transporter small subunit 1	SLC7A5	0,831	0,079
P78324	Tyrosine-protein phosphatase non-receptor type substrate 1	SIRPA	0,83	0,099
P51149	ras-related protein Rab-7a	RAB7A	0,828	0,243
P43007	neutral amino acid transporter A	SLC1A4	0,827	0,652
Q15388	Mitochondrial import receptor subunit TOM20 homolog	TOMM20	0,824	0,063
P17301	Integrin alpha-2	ITGA2	0,823	0,638
O00571	ATP-dependent RNA helicase DDX3X	DDX3X	0,823	0,058
Q92973	transportin-1	TNPO1	0,821	0,804
P06576	ATP synthase subunit beta, mitochondrial	ATP5B	0,817	0,630
Q99623	Prohibitin-2	PHB2	0,815	0,976
P11498	pyruvate carboxylase, mitochondrial	PC	0,81	0,338
Q9P035	Very-long-chain (3R)-3-hydroxyacyl-CoA dehydratase 3	PTPLAD1; HACD3	0,81	0,479
Q9NUM4	Transmembrane protein 106B	TMEM106B	0,808	0,929
P54760	Ephrin type-B receptor 4	EPHB4	0,807	nq
P08069	Insulin-like growth factor 1 receptor	IGF1R	0,801	0,439

Q9BTM1	Histone H2A.J	H2AFJ	0,801	0,017
P16615	Sarcoplasmic/endoplasmic reticulum calcium ATPase 2	ATP2A2	0,796	0,459
Q07954	prolow-density lipoprotein receptor-related protein 1	LRP1	0,795	0,292
P07237	Protein disulfide-isomerase	P4HB	0,794	0,995
P21796	voltage-dependent anion-selective channel protein 1	VDAC1	0,794	0,475
P55060	Exportin-2	CSE1L	0,793	0,250
P06733	alpha-enolase	ENO1	0,792	0,359
Q00610	Clathrin heavy chain 1	CLTC	0,791	0,860
P23528	Cofilin-1	CFL1	0,788	0,789
P23284	peptidyl-prolyl cis-trans isomerase B	PPIB	0,784	nq
P08729	Keratin, type II cytoskeletal 7	KRT7	0,784	0,361
P30825	High affinity cationic amino acid transporter 1	SLC7A1	0,781	0,700
Q15084	Protein disulfide-isomerase A6	PDIA6	0,78	0,304
P31327	Carbamoyl-phosphate synthase [ammonia], mitochondrial	CPS1	0,774	0,066
P21333	Filamin-A	FLNA	0,773	0,383
P13639	Elongation factor 2	EEF2	0,762	0,431
O43707	Alpha-actinin-4	ACTN4	0,759	0,833
Q13641	Trophoblast glycoprotein	TPBG	0,754	0,104
P68371	Tubulin beta-4B chain	TUBB4B	0,753	0,220
Q9BXB1	Leucine-rich repeat-containing G-protein coupled receptor 4	LGR4	0,753	0,542
P41440	Folate transporter 1	SLC19A1	0,752	0,722
P21926	CD9 antigen	CD9	0,751	0,557
P31943	Heterogeneous nuclear ribonucleoprotein H	HNRNPH1	0,75	0,389
P19256	Lymphocyte function-associated antigen 3	CD58	0,748	0,509
P08238	Heat shock protein HSP 90-beta	HSP90AB1	0,746	nq
P09758	Tumor-associated calcium signal transducer 2	TACSTD2	0,739	0,096

P25445	Tumor necrosis factor receptor superfamily member 6	FAS	0,739	0,998
Q13740	CD166 antigen	ALCAM	0,739	0,551
P06753	Tropomyosin alpha-3 chain	TPM3	0,729	0,505
Q29983	MHC class I polypeptide-related sequence A	MICA	0,719	nq
P08195	4F2 cell-surface antigen heavy chain	SLC3A2	0,715	0,501
O15431	High affinity copper uptake protein 1	SLC31A1	0,712	0,020
Q8TEX9	Importin-4	IPO4	0,712	0,009
Q6P4Q7	metal transporter CNNM4	CNNM4	0,709	0,731
P11166	Solute carrier family 2, facilitated glucose transporter member 1	SLC2A1	0,709	nq
Q9Y277	Voltage-dependent anion-selective channel protein 3	VDAC3	0,707	0,435
Q15758	Neutral amino acid transporter B(0)	SLC1A5	0,705	0,058
Q99523	Sortilin	SORT1	0,69	0,536
P52895	Aldo-keto reductase family 1 member C2	AKR1C2	0,676	0,190
P46782	40S ribosomal protein S5	RPS5	0,674	0,009
P07437	tubulin beta chain	TUBB	0,668	0,259
Q4KMQ2	Anoctamin-6	ANO6	0,667	nq
P25205	DNA replication licensing factor mcm3	MCM3	0,666	0,766
P53794	sodium/myo-inositol cotransporter	SLC5A3	0,665	0,222
Q8NFJ5	Retinoic acid-induced protein 3	GPRC5A	0,658	nq
Q5VZV1	Protein-lysine methyltransferase METTL21C	METTL21C	0,651	0,267
P04920	Anion exchange protein 2	SLC4A2	0,649	0,263
Q8NE01	Metal transporter CNNM3	CNNM3	0,647	0,250
P15328	Folate receptor alpha	FOLR1	0,627	0,383
P0C854	Putative cat eye syndrome critical region protein 9	CECR9	0,618	0,606
Q13443	disintegrin and metalloproteinase domain-containing protein 9	ADAM9	0,616	0,085
Q3ZCN5	Otogelin-like protein	OTOGL	0,614	0,362

P13797	Plastin-3	PLS3	0,613	0,109
P13726	tissue factor	F3	0,611	0,796
P55290	Cadherin-13	CDH13	0,599	nq
Q15417	Calponin-3	CNN3	0,591	0,074
P11586	C-1-tetrahydrofolate synthase, cytoplasmic	MTHFD1	0,586	0,131
P35052	Glypican-1	GPC1	0,584	0,046
Q96QD8	sodium-coupled neutral amino acid transporter 2	SLC38A2	0,584	nq
P07900	Heat shock protein HSP 90-alpha	HSP90AA1	0,557	0,026
P00558	phosphoglycerate kinase 1	PGK1	0,543	0,160
P23634	Plasma membrane calcium-transporting ATPase 4	ATP2B4	0,535	0,561
Q14444	Caprin-1	CAPRIN1	0,528	0,201
Q03167	Transforming growth factor beta receptor type 3	TGFBR3	0,527	0,012
P08195	4F2 cell-surface antigen heavy chain	SLC3A2	0,527	nq
Q9UIQ6	Leucyl-cystinyl aminopeptidase	LNPEP	0,516	0,073
P08571	Monocyte differentiation antigen CD14	CD14	0,503	0,393
Q9NZJ5	Eukaryotic translation initiation factor 2-alpha kinase 3	EIF2AK3	0,5	0,074
B7Z368	Uncharacterized protein C10orf142	LOC100130539; C10orf142	0,479	nq
O15061	Synemin	SYNM	0,459	0,283
Q96QK1	Vacuolar protein sorting-associated protein 35	VPS35	0,357	nq
Q8TCZ2	CD99 antigen-like protein 2	CD99L2	0,265	0,210

Accession	Description	Gene symbol	Abundance Ratio: (KO IFITM1, IFNγ) / (KO IFITM1, ctrl)	P-value
P30486	HLA class I histocompatibility antigen, B-48 alpha chain	HLA-B	4,839	0,001
P04222	HLA class I histocompatibility antigen, Cw-3 alpha chain	HLA-C	4,336	0,023

P42224	Signal transducer and activator of transcription 1-alpha/beta	STAT1	4,034	0,004
P30479	HLA class I histocompatibility antigen, B-41 alpha chain	HLA-B	4,023	0,009
P09603	Macrophage colony-stimulating factor 1	CSF1	2,908	0,195
P11279	Lysosome-associated membrane glycoprotein 1	LAMP1	2,773	0,389
P61769	Beta-2-microglobulin	B2M	2,605	0,005
P05362	Intercellular adhesion molecule 1	ICAM1	2,565	0,003
P15941	Mucin-1	MUC1	2,234	nq
Q99714	3-hydroxyacyl-CoA dehydrogenase type-2	HSD17B10	2,112	nq
Q9UGT4	Sushi domain-containing protein 2	SUSD2	2,004	nq
P22695	Cytochrome b-c1 complex subunit 2, mitochondrial	UQCRC2	1,963	0,106
Q8WVN6	Secreted and transmembrane protein 1	SECTM1	1,959	nq
P05534	HLA class I histocompatibility antigen, A-24 alpha chain	HLA-A	1,767	0,001
Q08722	Leukocyte surface antigen CD47	CD47	1,757	0,093
P25942	Tumor necrosis factor receptor superfamily member 5	CD40	1,734	0,364
Q10589	bone marrow stromal antigen 2	BST2	1,64	0,197
Q15262	Receptor-type tyrosine-protein phosphatase kappa	PTPRK	1,58	0,112
P18433	Receptor-type tyrosine-protein phosphatase alpha	PTPRA	1,569	nq
Q9NZJ5	Eukaryotic translation initiation factor 2-alpha kinase 3	EIF2AK3	1,554	0,409
Q9H5V8	CUB domain-containing protein 1	CDCP1	1,487	0,076
Q9BXB1	Leucine-rich repeat-containing G-protein coupled receptor 4	LGR4	1,41	0,358
Q9NV96	Cell cycle control protein 50A	TMEM30A	1,388	0,384
Q86X29	Lipolysis-stimulated lipoprotein receptor	LSR	1,381	0,628
Q5VZV1	Protein-lysine methyltransferase METTL21C	METTL21C	1,377	0,729
Q04912	Macrophage-stimulating protein receptor	MST1R	1,363	0,145
Q96NT5	Proton-coupled folate transporter	SLC46A1	1,354	0,543
P13164	Interferon-induced transmembrane protein 1	IFITM1	1,32	nq

O15427	Monocarboxylate transporter 4	SLC16A3	1,309	0,373
Q9Y289	Sodium-dependent multivitamin transporter	SLC5A6	1,301	0,147
P01130	Low-density lipoprotein receptor	LDLR	1,298	0,136
Q8TCZ2	CD99 antigen-like protein 2	CD99L2	1,294	0,393
Q16563	Synaptophysin-like protein 1	SYPL1	1,265	0,213
Q00610	Clathrin heavy chain 1	CLTC	1,262	nq
Q9P035	Very-long-chain (3R)-3-hydroxyacyl-CoA dehydratase 3	PTPLAD1; HACD3	1,261	0,613
Q687X5	Metalloreductase STEAP4	STEAP4	1,244	0,196
P25445	Tumor necrosis factor receptor superfamily member 6	FAS	1,239	nq
P04920	Anion exchange protein 2	SLC4A2	1,235	0,616
Q04941	proteolipid protein 2	PLP2	1,229	0,125
P04844	Dolichyl-diphosphooligosaccharide--protein glycosyltransferase subunit 2	RPN2	1,228	0,676
Q9NPH3	Interleukin-1 receptor accessory protein	IL1RAP	1,214	0,974
P07204	Thrombomodulin	THBD	1,214	nq
P08581	Hepatocyte growth factor receptor	MET	1,214	0,192
P17813	Endoglin	ENG	1,21	0,464
Q3ZCN5	Otogelin-like protein	OTOGL	1,207	nq
Q9Y6M7	Sodium bicarbonate cotransporter 3	SLC4A7	1,185	0,743
Q9Y639	Neuroplastin	NPTN	1,175	0,925
Q9UNN8	Endothelial protein C receptor	PROCR	1,165	0,920
P49184	Deoxyribonuclease-1-like 1	DNASE1L1	1,159	0,525
P09622	Dihydrolipoyl dehydrogenase, mitochondrial	DLD	1,154	nq
P63000	Ras-related C3 botulinum toxin substrate 1	RAC1	1,149	0,490
P07099	epoxide hydrolase 1	EPHX1	1,135	0,901
Q6P4Q7	metal transporter CNNM4	CNNM4	1,135	0,240
Q969P0	Immunoglobulin superfamily member 8	IGSF8	1,132	0,035

P27824	Calnexin	CANX	1,123	0,256
P30153	serine/threonine-protein phosphatase 2A 65 kDa regulatory subunit A alpha isoform	PPP2R1A	1,122	0,590
P21926	CD9 antigen	CD9	1,121	0,908
P62979	Ubiquitin-40S ribosomal protein S27a	RPS27A	1,106	0,777
P29323	Ephrin type-B receptor 2	EPHB2	1,104	0,811
P60033	CD81 antigen	CD81	1,102	0,251
Q8NE01	Metal transporter CNNM3	CNNM3	1,1	0,560
P04843	Dolichyl-diphosphooligosaccharide--protein glycosyltransferase subunit 1	RPN1	1,099	0,890
Q92542	Nicastrin	NCSTN	1,099	0,442
P27797	Calreticulin	CALR	1,093	0,871
Q4KMQ2	Anoctamin-6	ANO6	1,089	0,508
P15529	Membrane cofactor protein	CD46	1,078	0,157
Q15758	Neutral amino acid transporter B(0)	SLC1A5	1,073	0,348
A5PL33	protein KRBA1	KRBA1	1,07	0,962
P05141	ADP/ATP translocase 2	SLC25A5	1,067	0,857
O75144	ICOS ligand	ICOSLG; LOC102723996	1,067	0,558
P23284	peptidyl-prolyl cis-trans isomerase B	PPIB	1,06	0,687
P18084	Integrin beta-5	ITGB5	1,049	0,219
Q8IWT6	volume-regulated anion channel subunit LRRC8A	LRRC8A	1,045	0,339
P04626	Receptor tyrosine-protein kinase erbB-2	ERBB2	1,042	0,602
Q9Y6M5	zinc transporter 1	SLC30A1	1,032	0,486
P05026	Sodium/potassium-transporting ATPase subunit beta-1	ATP1B1	1,03	0,143
Q6EMK4	vasorin	VASN	1,026	0,839
P43121	Cell surface glycoprotein MUC18	MCAM	1,015	0,946

Q15084	Protein disulfide-isomerase A6	PDIA6	1,009	0,887
P08962	CD63 antigen	CD63	1,007	0,870
Q9Y666	Solute carrier family 12 member 7	SLC12A7	1,006	nq
O95297	Myelin protein zero-like protein 1	MPZL1	1,001	0,846
P16144	Integrin beta-4	ITGB4	0,996	0,904
P63261	Actin, cytoplasmic 2	ACTG1	0,99	0,887
P48509	CD151 antigen	CD151	0,989	0,995
Q13421	Mesothelin	MSLN	0,989	0,860
P04899	Guanine nucleotide-binding protein G(i) subunit alpha-2	GNAI2	0,987	0,813
P09382	Galectin-1	LGALS1	0,987	0,708
P21796	voltage-dependent anion-selective channel protein 1	VDAC1	0,987	0,865
Q6NSJ0	Uncharacterized family 31 glucosidase KIAA1161	KIAA1161; MYORG	0,986	0,971
P11717	Cation-independent mannose-6-phosphate receptor	IGF2R	0,976	0,536
P16070	CD44 antigen	CD44	0,967	0,639
Q15417	Calponin-3	CNN3	0,96	0,894
P55290	Cadherin-13	CDH13	0,959	0,750
B7Z368	Uncharacterized protein C10orf142	LOC100130539; C10orf142	0,956	0,917
Q8IWA5	Choline transporter-like protein 2	SLC44A2	0,956	0,427
P25705	ATP synthase subunit alpha, mitochondrial	ATP5A1	0,955	0,470
Q03405	Urokinase plasminogen activator surface receptor	PLAUR	0,954	0,728
Q7L1W4	volume-regulated anion channel subunit LRRC8D	LRRC8D	0,953	0,789
P78324	Tyrosine-protein phosphatase non-receptor type substrate 1	SIRPA	0,952	0,448
P14314	Glucosidase 2 subunit beta	PRKCSH	0,952	0,718
P35613	Basigin	BSG	0,951	0,529
P15151	Poliovirus receptor	PVR	0,948	0,984

O00592	Podocalyxin	PODXL	0,944	0,532
P29317	Ephrin type-A receptor 2	EPHA2	0,943	0,936
Q5RHP9	glutamate-rich protein 3	C1orf173; ERICH3	0,933	0,397
P33527	Multidrug resistance-associated protein 1	ABCC1	0,932	0,419
Q96QD8	sodium-coupled neutral amino acid transporter 2	SLC38A2	0,932	0,966
Q13740	CD166 antigen	ALCAM	0,932	nq
P23634	Plasma membrane calcium-transporting ATPase 4	ATP2B4	0,931	0,633
P05023	Sodium/potassium-transporting ATPase subunit alpha-1	ATP1A1	0,931	0,624
Q15388	Mitochondrial import receptor subunit TOM20 homolog	TOMM20	0,928	0,799
Q8WTV0	Scavenger receptor class B member 1	SCARB1	0,928	0,935
O00571	ATP-dependent RNA helicase DDX3X	DDX3X	0,927	0,436
P32004	Neural cell adhesion molecule L1	L1CAM	0,925	0,909
P15328	Folate receptor alpha	FOLR1	0,924	nq
P48960	CD97 antigen	CD97; ADGRE5	0,924	0,923
Q00325	Phosphate carrier protein, mitochondrial	SLC25A3	0,919	0,436
Q02978	Mitochondrial 2-oxoglutarate/malate carrier protein	SLC25A11	0,918	nq
Q12913	Receptor-type tyrosine-protein phosphatase eta	PTPRJ	0,917	0,107
Q6YHK3	CD109 antigen	CD109	0,917	0,520
Q01650	large neutral amino acids transporter small subunit 1	SLC7A5	0,916	0,896
Q9H2H9	Sodium-coupled neutral amino acid transporter 1	SLC38A1	0,914	0,809
P54709	sodium/potassium-transporting ATPase subunit beta-3	ATP1B3	0,914	0,873
P11586	C-1-tetrahydrofolate synthase, cytoplasmic	MTHFD1	0,913	0,695
Q15043	Zinc transporter ZIP14	SLC39A14	0,911	0,518
P31327	Carbamoyl-phosphate synthase [ammonia], mitochondrial	CPS1	0,907	0,910
P53794	sodium/myo-inositol cotransporter	SLC5A3	0,904	0,790
P06576	ATP synthase subunit beta, mitochondrial	ATP5B	0,903	0,375

Q92692	Nectin-2	PVRL2; NECTIN2	0,902	0,739
P14384	Carboxypeptidase M	CPM	0,9	0,638
P30101	Protein disulfide-isomerase A3	PDIA3	0,898	0,887
P23396	40S ribosomal protein S3	RPS3	0,897	0,830
P50895	Basal cell adhesion molecule	BCAM	0,891	0,857
Q96J84	Kin of IRRE-like protein 1	KIRREL; KIRREL1	0,889	0,398
P49915	GMP synthase [glutamine-hydrolyzing]	GMPS	0,889	0,695
P08195	4F2 cell-surface antigen heavy chain	SLC3A2	0,887	0,652
Q9NUM4	Transmembrane protein 106B	TMEM106B	0,885	0,791
P11021	78 kDa glucose-regulated protein	HSPA5	0,881	0,645
P00533	epidermal growth factor receptor	EGFR	0,881	0,376
P02786	Transferrin receptor protein 1	TFRC	0,88	0,656
P26006	Integrin alpha-3	ITGA3	0,88	0,644
P11498	pyruvate carboxylase, mitochondrial	PC	0,878	0,735
Q13443	disintegrin and metalloproteinase domain-containing protein 9	ADAM9	0,873	0,677
P10809	60 kDa heat shock protein, mitochondrial	HSPD1	0,872	0,557
Q5ZPR3	CD276 antigen	CD276	0,872	0,347
Q13308	Inactive tyrosine-protein kinase 7	PTK7	0,87	0,992
O15031	Plexin-B2	PLXNB2	0,87	0,697
P08069	Insulin-like growth factor 1 receptor	IGF1R	0,87	0,987
P39656	Dolichyl-diphosphooligosaccharide--protein glycosyltransferase 48 kDa subunit	DDOST	0,869	0,876
P06756	Integrin alpha-V	ITGAV	0,868	0,444
Q9UIQ6	Leucyl-cystinyl aminopeptidase	LNPEP	0,868	0,866
P62937	peptidyl-prolyl cis-trans isomerase A	PPIA	0,868	0,770
P13987	CD59 glycoprotein	CD59	0,866	0,881

Q9UIW2	Plexin-A1	PLXNA1	0,866	0,363
P14625	Endoplasmin	HSP90B1	0,864	0,925
P06753	Tropomyosin alpha-3 chain	TPM3	0,858	0,583
Q9BZM5	UL16-binding protein 2	ULBP2	0,856	0,926
Q13162	Peroxiredoxin-4	PRDX4	0,854	0,718
Q92859	neogenin	NEO1	0,849	0,517
P13667	Protein disulfide-isomerase A4	PDIA4	0,849	0,925
P10586	Receptor-type tyrosine-protein phosphatase F	PTPRF	0,848	0,199
P20827	Ephrin-A1	EFNA1	0,848	0,563
P05556	Integrin beta-1	ITGB1	0,846	0,567
P0C854	Putative cat eye syndrome critical region protein 9	CECR9	0,843	0,501
Q8NFZ8	Cell adhesion molecule 4	CADM4	0,842	0,992
O14672	Disintegrin and metalloproteinase domain-containing protein 10	ADAM10	0,842	0,420
P23229	integrin alpha-6	ITGA6	0,841	0,869
P08195	4F2 cell-surface antigen heavy chain	SLC3A2	0,841	0,791
Q99650	Oncostatin-M-specific receptor subunit beta	OSMR	0,837	0,511
P14209	CD99 antigen	CD99	0,837	0,500
Q14697	Neutral alpha-glucosidase AB	GANAB	0,834	0,855
P14923	Junction plakoglobin	JUP	0,833	0,813
P50454	Serpin H1	SERPINH1	0,831	0,451
P98172	ephrin-B1	EFNB1	0,831	0,334
P54760	Ephrin type-B receptor 4	EPHB4	0,831	0,286
Q14118	Dystroglycan	DAG1	0,824	0,674
P53985	Monocarboxylate transporter 1	SLC16A1	0,822	0,136
Q06830	peroxiredoxin-1	PRDX1	0,816	0,369
O43278	Kunitz-type protease inhibitor 1	SPINT1	0,814	0,288

Q9P2B2	prostaglandin F2 receptor negative regulator	PTGFRN	0,813	0,244
Q03167	Transforming growth factor beta receptor type 3	TGFBR3	0,812	0,728
Q9ULI3	Protein HEG homolog 1	HEG1	0,811	0,182
Q99523	Sortilin	SORT1	0,811	0,288
P23528	Cofilin-1	CFL1	0,808	0,700
O14944	Proepiregulin	EREG	0,807	0,838
Q14126	Desmoglein-2	DSG2	0,806	0,935
P30041	Peroxiredoxin-6	PRDX6	0,802	0,252
P38646	Stress-70 protein, mitochondrial	HSPA9	0,801	0,657
P42704	Leucine-rich PPR motif-containing protein, mitochondrial	LRPPRC	0,8	0,918
P20645	Cation-dependent mannose-6-phosphate receptor	M6PR	0,799	nq
O15431	High affinity copper uptake protein 1	SLC31A1	0,798	0,121
Q8TB96	T-cell immunomodulatory protein	ITFG1	0,798	0,498
Q9BTM1	Histone H2A.J	H2AFJ	0,796	0,665
P41440	Folate transporter 1	SLC19A1	0,794	0,294
P06703	protein S100-A6	S100A6	0,791	0,291
Q04721	Neurogenic locus notch homolog protein 2	NOTCH2	0,79	0,841
P05187	alkaline phosphatase, placental type	ALPP	0,788	0,155
O15061	Synemin	SYNM	0,788	0,479
P11166	Solute carrier family 2, facilitated glucose transporter member 1	SLC2A1	0,785	0,465
P09758	Tumor-associated calcium signal transducer 2	TACSTD2	0,785	0,548
P45880	Voltage-dependent anion-selective channel protein 2	VDAC2	0,783	0,592
Q99623	Prohibitin-2	PHB2	0,782	0,691
P30530	Tyrosine-protein kinase receptor UFO	AXL	0,778	0,474
Q13332	Receptor-type tyrosine-protein phosphatase S	PTPRS	0,775	0,773
P42892	Endothelin-converting enzyme 1	ECE1	0,77	0,838

O15230	Laminin subunit alpha-5	LAMA5	0,765	0,200
Q15365	Poly(RC)-binding protein 1	PCBP1	0,765	0,317
Q13641	Trophoblast glycoprotein	TPBG	0,764	0,670
P31431	syndecan-4	SDC4	0,761	0,161
P56199	Integrin alpha-1	ITGA1	0,761	0,825
O14763	Tumor necrosis factor receptor superfamily member 10B	TNFRSF10B	0,759	0,183
Q8WWI5	choline transporter-like protein 1	SLC44A1	0,754	nq
P19256	Lymphocyte function-associated antigen 3	CD58	0,751	0,282
Q07954	prolow-density lipoprotein receptor-related protein 1	LRP1	0,75	0,576
P08648	Integrin alpha-5	ITGA5	0,75	0,829
P62826	GTP-binding nuclear protein RAN	RAN	0,748	0,090
P47756	F-actin-capping protein subunit beta	CAPZB	0,747	0,228
P07437	tubulin beta chain	TUBB	0,745	0,221
Q9H6X2	Anthrax toxin receptor 1	ANTXR1	0,741	0,662
Q12907	Vesicular integral-membrane protein VIP36	LMAN2	0,738	0,514
P15529	Membrane cofactor protein	CD46	0,738	0,198
P48047	ATP synthase subunit O, mitochondrial	ATP5O	0,736	0,473
P68363	Tubulin alpha-1B chain	TUBA1B	0,734	0,310
P31641	Sodium- and chloride-dependent taurine transporter	SLC6A6	0,732	0,270
Q04695	Keratin, type I cytoskeletal 17	KRT17	0,73	0,514
P35232	Prohibitin	PHB	0,725	0,805
P18827	syndecan-1	SDC1	0,721	0,325
Q92841	Probable ATP-dependent RNA helicase DDX17	DDX17	0,719	0,129
Q99497	protein/nucleic acid deglycase DJ-1	PARK7	0,716	nq
P49411	elongation factor Tu, mitochondrial	TUFM	0,715	0,003
P43007	neutral amino acid transporter A	SLC1A4	0,714	0,978

P68104	Elongation factor 1-alpha 1	EEF1A1	0,704	0,169
P60709	Actin, cytoplasmic 1	ACTB	0,703	0,244
P46940	Ras GTPase-activating-like protein IQGAP1	IQGAP1	0,698	0,271
P07237	Protein disulfide-isomerase	P4HB	0,698	0,665
Q6PL18	ATPase family AAA domain-containing protein 2	ATAD2	0,697	0,501
P13797	Plastin-3	PLS3	0,696	0,367
Q9Y624	Junctional adhesion molecule A	F11R	0,693	0,328
P07355	Annexin A2	ANXA2	0,69	0,266
P08754	Guanine nucleotide-binding protein G(k) subunit alpha	GNAI3	0,689	0,037
P30825	High affinity cationic amino acid transporter 1	SLC7A1	0,688	0,025
P68371	Tubulin beta-4B chain	TUBB4B	0,678	0,193
P08174	Complement decay-accelerating factor	CD55	0,677	0,184
Q12931	heat shock protein 75 kDa, mitochondrial	TRAP1	0,675	nq
P11142	Heat shock cognate 71 kDa protein	HSPA8	0,674	0,262
P17342	Atrial natriuretic peptide receptor 3	NPR3	0,673	0,736
P49368	T-complex protein 1 subunit gamma	CCT3	0,673	0,242
Q9Y2B0	Protein canopy homolog 2	CNPY2	0,672	0,551
P78310	Coxsackievirus and adenovirus receptor	CXADR	0,671	0,557
P35052	Glypican-1	GPC1	0,669	0,336
P17301	Integrin alpha-2	ITGA2	0,667	0,640
Q8NFJ5	Retinoic acid-induced protein 3	GPRC5A	0,664	0,535
Q86V24	Adiponectin receptor protein 2	ADIPOR2	0,657	0,283
P16615	Sarcoplasmic/endoplasmic reticulum calcium ATPase 2	ATP2A2	0,656	0,331
P60981	Destrin	DSTN	0,655	0,152
Q13444	Disintegrin and metalloproteinase domain-containing protein 15	ADAM15	0,653	0,110
P31946	14-3-3 protein beta/alpha	YWHAB	0,65	0,429

Q92673	Sortilin-related receptor	SORL1	0,646	0,388
P13726	tissue factor	F3	0,642	0,197
P78527	DNA-dependent protein kinase catalytic subunit	PRKDC	0,638	0,440
P51149	ras-related protein Rab-7a	RAB7A	0,635	0,907
Q15149	plectin	PLEC	0,633	0,277
P08729	Keratin, type II cytoskeletal 7	KRT7	0,628	0,229
P55060	Exportin-2	CSE1L	0,624	0,244
P12645	bone morphogenetic protein 3	BMP3	0,622	0,038
P13473	Lysosome-associated membrane glycoprotein 2	LAMP2	0,621	nq
P50281	Matrix metalloproteinase-14	MMP14	0,619	0,425
P43307	Translocon-associated protein subunit alpha	SSR1	0,617	0,475
P08238	Heat shock protein HSP 90-beta	HSP90AB1	0,615	0,264
O75153	Clustered mitochondria protein homolog	CLUH; KIAA0664	0,615	0,230
P04156	Major prion protein	PRNP	0,613	0,196
P78380	Oxidized low-density lipoprotein receptor 1	OLR1	0,612	nq
O75369	Filamin-B	FLNB	0,609	0,425
P14618	Pyruvate kinase PKM	PKM	0,598	0,190
Q92973	transportin-1	TNPO1	0,598	0,363
O43707	Alpha-actinin-4	ACTN4	0,595	0,201
P30048	Thioredoxin-dependent peroxide reductase, mitochondrial	PRDX3	0,591	0,585
Q9NQC3	Reticulon-4	RTN4	0,578	0,042
P0DMV8	heat shock 70 kDa protein 1A	HSPA1A; HSPA1B	0,564	0,175
P08758	annexin A5	ANXA5	0,564	0,232
P13639	Elongation factor 2	EEF2	0,563	0,222
P04406	glyceraldehyde-3-phosphate dehydrogenase	GAPDH	0,563	0,281
P07900	Heat shock protein HSP 90-alpha	HSP90AA1	0,557	0,257

P50990	T-complex protein 1 subunit theta	CCT8	0,554	0,433
Q14240	Eukaryotic initiation factor 4A-II	EIF4A2	0,554	0,338
Q92598	Heat shock protein 105 kDa	HSPH1	0,53	0,154
Q16891	MICOS complex subunit Mic60	IMMT	0,51	0,180
P21333	Filamin-A	FLNA	0,508	0,365
Q9Y277	Voltage-dependent anion-selective channel protein 3	VDAC3	0,501	0,053
P07737	profilin-1	PFN1	0,499	0,229
P05787	Keratin, type II cytoskeletal 8	KRT8	0,499	0,555
Q29983	MHC class I polypeptide-related sequence A	MICA	0,488	0,408
P22314	Ubiquitin-like modifier-activating enzyme 1	UBA1	0,484	0,147
Q8TEX9	Importin-4	IPO4	0,461	0,341
P61978	Heterogeneous nuclear ribonucleoprotein K	HNRNPK	0,457	nq
P30086	phosphatidylethanolamine-binding protein 1	PEBP1	0,455	0,476
P31943	Heterogeneous nuclear ribonucleoprotein H	HNRNPH1	0,454	0,338
P00558	phosphoglycerate kinase 1	PGK1	0,445	0,173
P52895	Aldo-keto reductase family 1 member C2	AKR1C2	0,438	0,417
Q14444	Caprin-1	CAPRIN1	0,407	nq
P32119	Peroxiredoxin-2	PRDX2	0,401	0,374
P06733	alpha-enolase	ENO1	0,398	0,215
Q9UJS0	Calcium-binding mitochondrial carrier protein Aralar2	SLC25A13	0,374	0,247
Q00341	Vigilin	HDLBP	0,358	nq
P52272	Heterogeneous nuclear ribonucleoprotein M	HNRNPM	0,33	0,247
P25205	DNA replication licensing factor mcm3	MCM3	0,298	0,407
Q96QK1	Vacuolar protein sorting-associated protein 35	VPS35	0,288	nq
