# Supplementary material for: IFITM1 as a modulator of surfaceome dynamics and aggressive phenotype in cervical cancer cells
Source: Oncol Rep. 2025 Apr 29;53(6):71. doi: 10.3892/or.2025.8904 (PMC12059461; doi:10.3892/or.2025.8904)

Figure S1. FACS gating strategy for the NK cytotoxicity assay. Using the P1 gate, live and dead cells were selected from all events. The P3 gate was used to select singlet cells from the P1 population. The cell populations were separated on the basis of the CFSE signal detected in the FITC channel; specifically, CFSE-positive events (P4 gate) indicated tumor (SiHa) cells, and CFSE-negative events were considered NK92 cells. Dead tumor cells were analyzed within the P4 population and distinguished on the basis of 7-AAD staining (P6 gate).

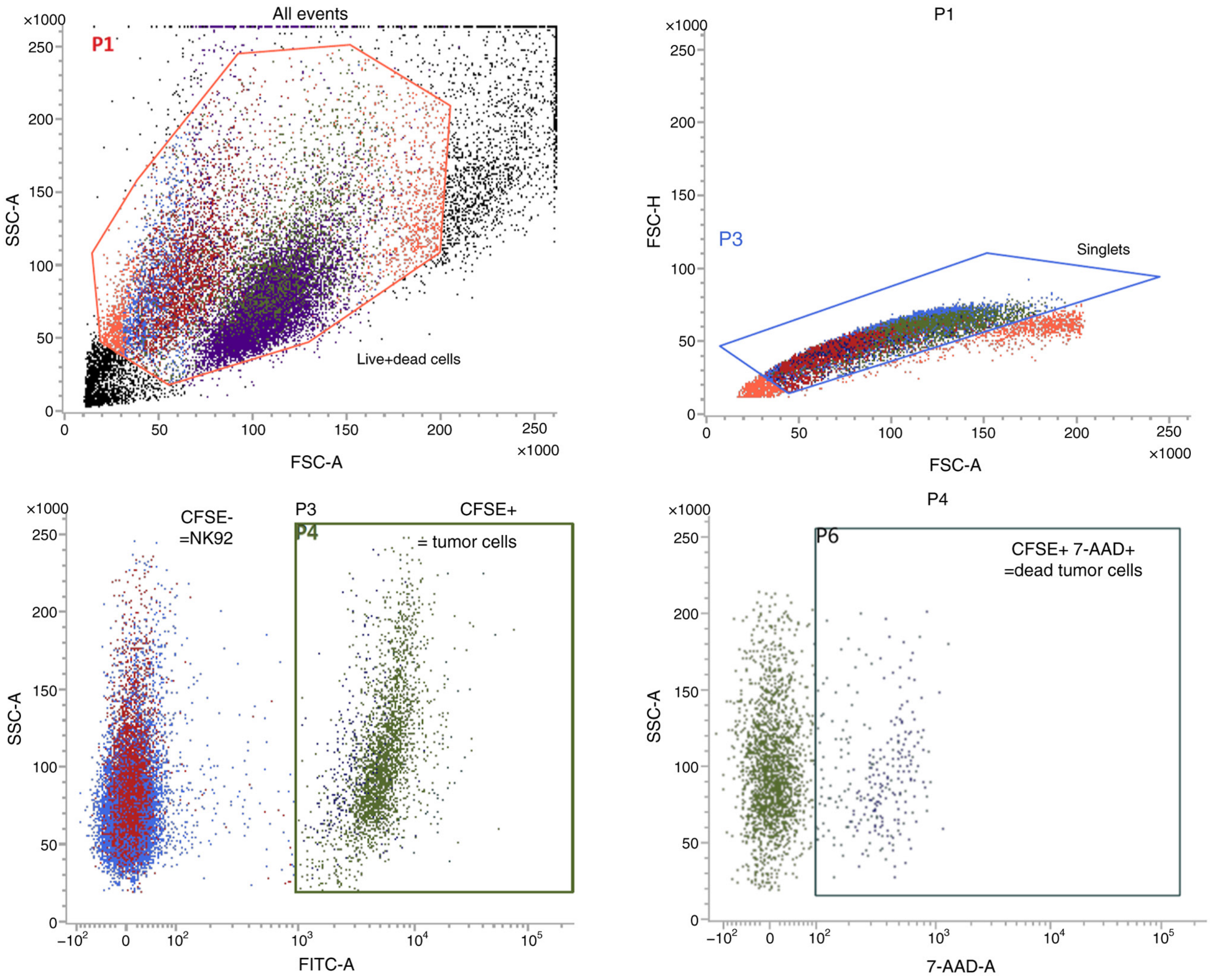

Figure S2. Peptide abundances indicating proteins with differential membrane levels between SiHa wt and SiHa IFITM1 KO cells and the effect of IFNγ. The error bars represent the mean ± SEM from biological replicates. *P<0.05, **P<0.01 and ***P<0.001 when comparing wt and IFITM1 KO; #P<0.05, ##P<0.01 when comparing CTRL and respective IFNγ-treated samples. wt, wild-type; KO, knockout; LNPEP, leucyl and cystinyl aminopeptidase; IFITM, interferon-induced transmembrane protein; IFN, interferon; wt, wild-type; KO, knockout; CTRL, control.

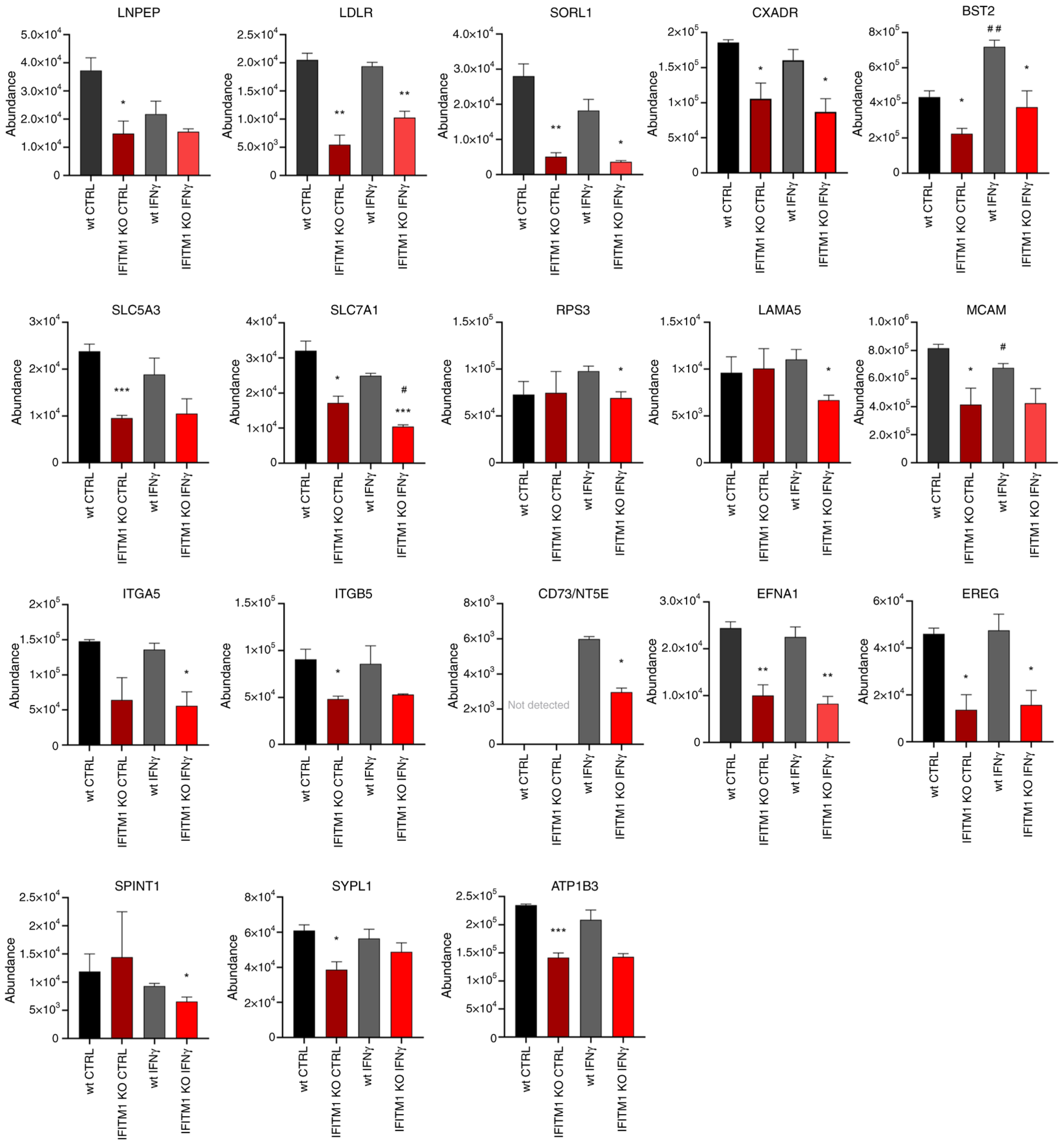

Figure S3. FACS analysis-representative data. (A) CD166 and CD40 cell surface abundance in SiHa IFITM1/3 KO clones and controls (wt, scrambled) shown as dot plots of individual cell samples. The numbers indicate the percentage of positive cell populations, with gating set based on unstained control (ctrl) samples. (B) Detection of dead SiHa IFITM1/3 KO cells and controls (7-AAD+ within the CFSE+ population) after 4 h of co-culture with NK cells. Representative dot plots display the percentage of dead SiHa cells cultured alone (SiHa only, upper panels) or co-cultured with NK92 cells at a 10:1 ratio (NK92: SiHa, bottom panels).

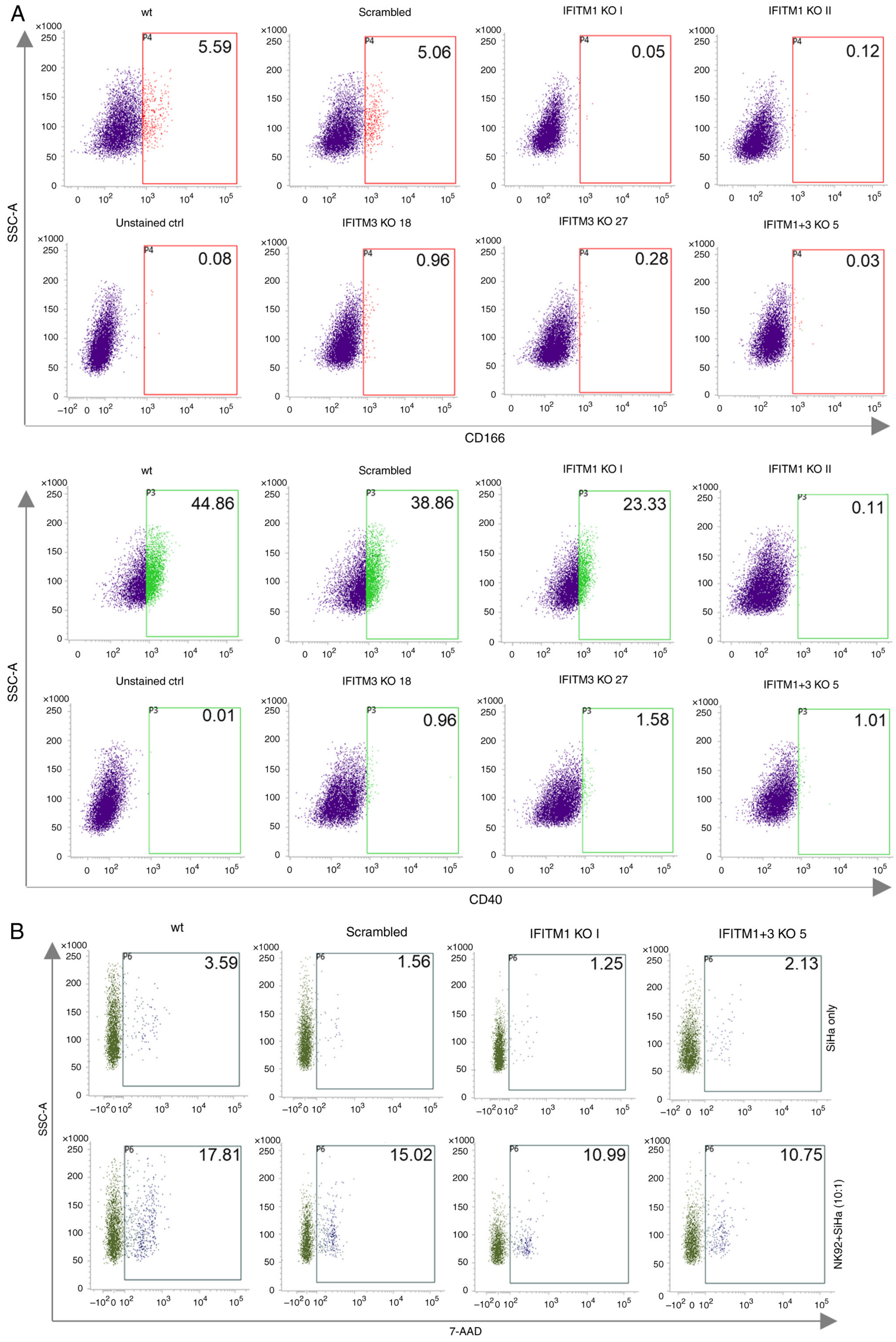

Supplement: Supporting Data [file Supplementary_Data2.pdf]
